# Conformation of Pyroglutamated Amyloid β (3–40) and (11–40) Fibrils – Extended or Hairpin?

**DOI:** 10.1021/acs.jpcb.3c07285

**Published:** 2024-02-09

**Authors:** Holger A. Scheidt, Alexander Korn, Benedikt Schwarze, Martin Krueger, Daniel Huster

**Affiliations:** † Institute for Medical Physics and Biophysics, Leipzig University Härtelstr. 16/18, D-04107 Leipzig, Germany; ‡ Institute of Anatomy, Leipzig University, Liebigstr. 13, 04103 Leipzig, Germany

## Abstract

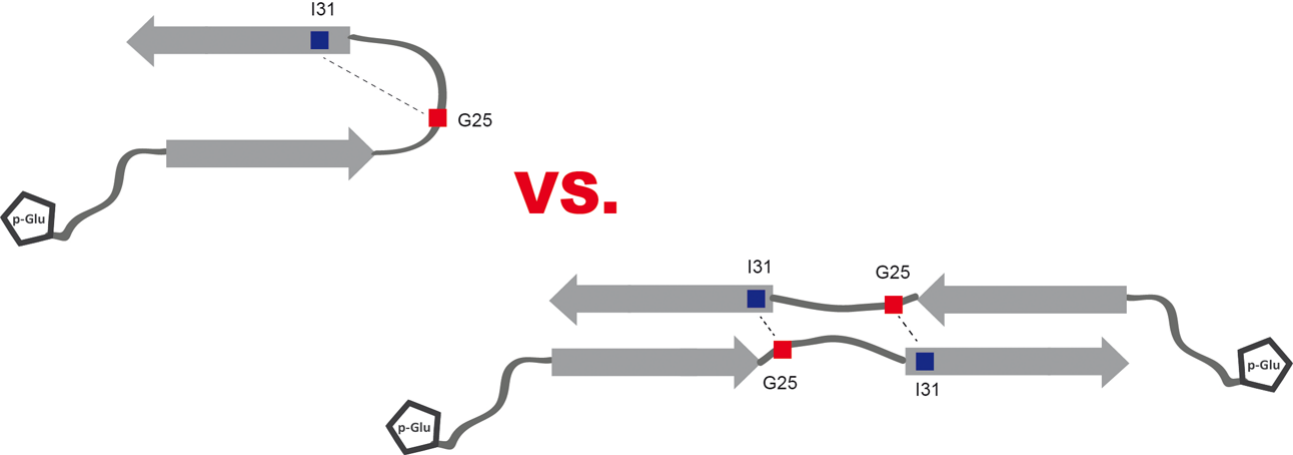

Amyloid β (Aβ) is a hallmark protein of Alzheimer‘s disease. One physiologically important Aβ variant is formed by initial N-terminal truncation at a glutamic acid position (either E_3_ or E_11_), which is subsequently cyclized to a pyroglutamate (either pE_3_ or pE_11_). Both forms have been found in high concentrations in the core of amyloid plaques and are likely of high importance in the pathology of Alzheimer’s disease. However, the molecular structure of the fibrils of these variants is not entirely clear. Solid-state NMR spectroscopy studies have reported a molecular contact between Gly_25_ and Ile_31_, which would disagree with the conventional hairpin model of wildtype (WT-)Aβ_1–40_ fibrils, most often described in the literature. We investigated the conformation of the monomeric unit of pE_3_-Aβ_3–40_ and pE_11_-Aβ_11–40_ (and for comparison also wildtype (WT)-Aβ_1–40_) fibrils to find out whether the hairpin or a newly suggested extended structure dominates the structure of the Aβ monomers in these fibrils. To this end, solid-state NMR spectroscopy was applied probing the inter-residual contacts between Phe_19_/Leu_34_, Ala_21_/Leu_34_, and especially Gly_25_/Ile_31_ using suitable isotopic labeling schemes. In the second part, the flexible turn of the Aβ_40_ peptides was replaced by a (3-(3-aminomethyl)phenylazo)phenylacetic acid (AMPP)-based photoswitch, which can predefine the peptide conformation to either an extended (*trans*) or hairpin (*cis*) conformation. This enables simultaneous spectroscopic assessment of the conformation of the AMPP-photoswitch, allowing in situ structural investigations during fibrillation in contrast to structural techniques such as NMR spectroscopy or cryo-EM, which can only be applied to stable conformers. Both methods confirm an extended structure for the peptidic monomers in fibrils of all investigated Aβ variants. Especially the Gly_25_/Ile_31_ contact is a decisive indicator for the extended structure along with the characteristic absorption spectra of *trans*-AMPP-Aβ.

## Introduction

Chemical modifications of biomolecules are key to biological function but also malfunction, leading to pathologies. For proteins, numerous posttranslational modifications have been reported.^1^ The senile plaques in the brains of Alzheimer’s patients are formed by amyloid β (Aβ) peptides of varying lengths.^2,3^ While the aggregation pathways, structure, and dynamics of the most abundant Aβ species with 40 (Aβ_40_) or 42 (Aβ_42_) residues have been extensively studied for many years,^4,5^ naturally truncated and/or further post-translationally modified Aβ peptides have only been discussed with regard to their role in the pathology of Alzheimer’s disease in the past decade.^6−8^ For instance, N-terminally truncated Aβ peptides featuring a glutamate residue in position 3 or 11 are further processed by glutaminyl cyclase to form a cyclic pyroglutamate at the N-terminus.^9^ These chemically modified variants show not only much faster fibrillation kinetics but also higher cytotoxicity.^10−14^ Furthermore, it was found that the concentration of both peptide variants (pE_3_-Aβ and pE_11_-Aβ) in the core of amyloid plaques was higher suggesting that they are capable of initiating aggregation.^15^ In a rodent model of AD, inhibiting the glutaminyl cyclase resulted in improved performance in context memory and spatial learning tests, which suggests that the cyclization may be a very important determinant in the development of the disease.^9^ Therefore, pyroglutamate modified Aβ as well as the glutaminyl cyclase are targets in current drug development efforts.^16^

**Figure 1:**
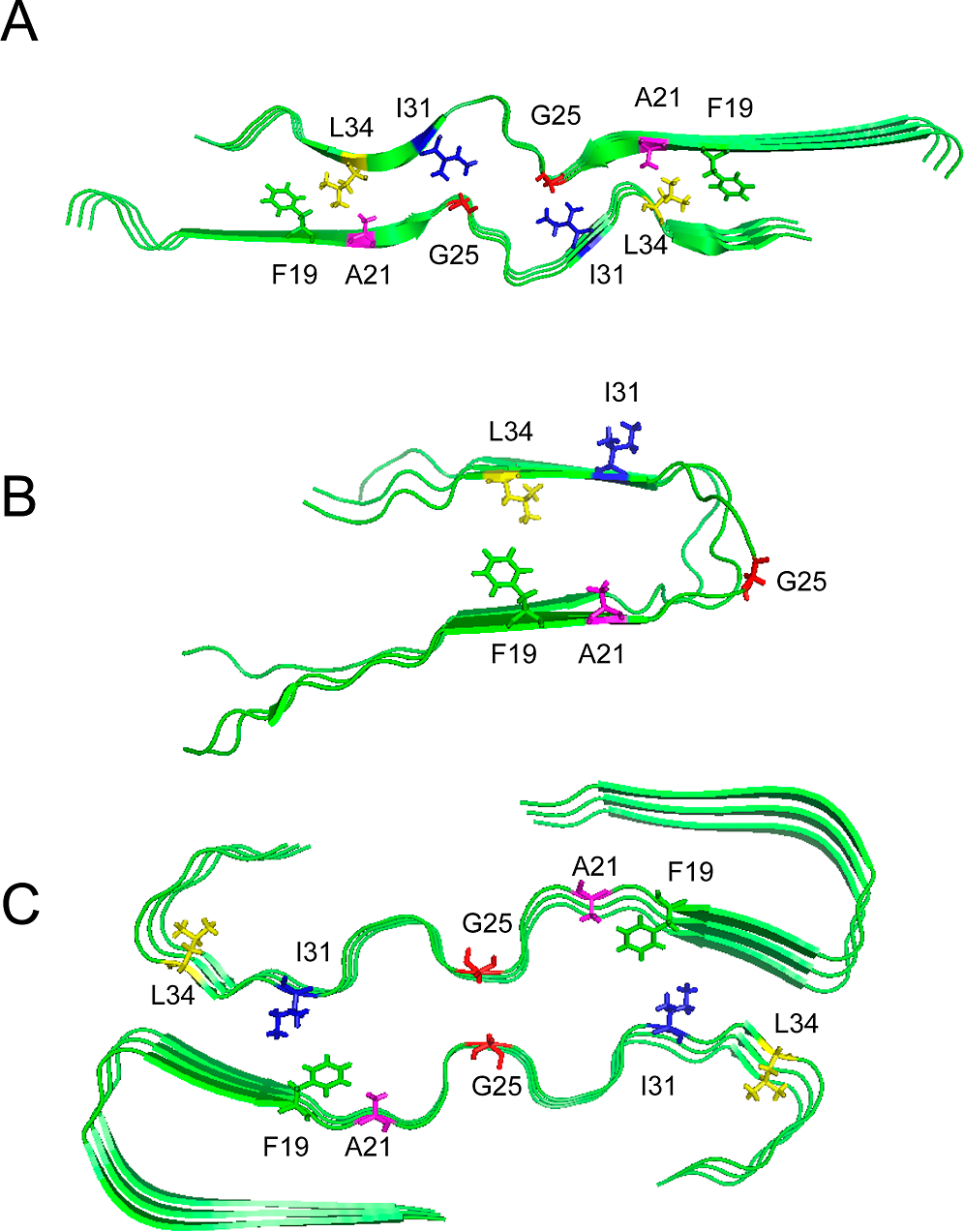
(A) The extended structural model for WT-Aβ_1–40_ (pdb 6W0O),^33^ (B) one of the different hairpin models (pdb 2LMN),^26^ and (C) a cryo-EM model (pdb 6SHS).^41^ The residues investigated in this study are color-coded as follows: Phe_19_ (green), Ala_21_ (magenta), Gly_25_ (red), Ile_31_ (blue), and Leu_34_ (yellow).

The structure of fibrils, formed by these pathologically highly relevant Aβ variants, has attracted attention.^17−23^ In a recent solid-state NMR structural investigation of mature pE_11_-Aβ_11–40_ fibrils,^21^ molecular features were revealed that by and large, agreed with the known structural models of Aβ_1–40_. However, the same study also discovered a previously undescribed intermolecular contact between residues Gly_25_ and Ile_31_. This contact did not fit into any of the hairpin structural models for wildtype (WT) Aβ_1–40_,^24−29^ Aβ_1–42_ or other Aβ variants,^30−32^ which were available at that time. The reason was that the distance between these two amino acids in the models was too large to explain the detected cross peaks in the 2D ^13^C–^13^C NMR correlation spectra (Figure 1). In this regard, it is of high relevance that recently a new structural model for fibrils formed from WT-Aβ_1–40_ peptides was published by the Tycko lab,^33^ which for the first time exhibited a predominantly extended peptide structure in the core of the fibril (Figure 1) prominently showing this intermolecular contact between residues Gly_25_ and Ile_31_.

While the Gly_25_–Ile_31_ contact seems to be a unique interaction in this new extended model, it is important to note that this model also involves contacts so far assumed to be indicative of a hairpin conformation. For instance, the residues Phe_19_ and Leu_34_, which are extensively described as potentially both intra-^27^ or intermolecular contacts^26,34−39^ in different hairpin models (Figure 1A). The same holds true for Ala_21_ and Leu_34_, which were identified as intramolecular contact in a slightly different hairpin model by the Bertini laboratory.^28^ On the other hand, Ala_21_–Leu_34_ was observed as an intermolecular contact in fibrils formed from wildtype Aβ_1–40_, which were seeded with pE_3_-Aβ_3–40_ or truncated Aβ_3–40_^40^ described with an extended model. Noteworthy, in a recent cryo-EM structure of Aβ_1–40_ fibrils,^41^ which differs from the other models, none of the aforementioned molecular contacts seem possible (Figure 1C). All these different structural models demonstrate the known structural polymorphism of Aβ fibrils.^25,26,41^ In summary, upon comparison of all available pdb-structures for Aβ_1–40_, if present, the Gly_25_–Ile_31_ contact was identified as an intermolecular interaction unique for an extended structure of Aβ peptides. In addition, also the other contacts discussed (Phe_19_/Leu_34_, Ala_21_/Leu_34_) have to be confirmed as intermolecular to support this structural model.

The current study aims to assign both pyroglutamated variants pE_3_-Aβ_3–40_ and pE_11_-Aβ_11–40_ to one of the known structural models of Aβ fibrils and addresses the question of which model represents the predominant structure of these species under our experimental conditions. The results will shed light on the questions if the extended structure model, which can explain the previously observed Gly_25_–Ile_31_ contact, is relevant also for the pyroglutamated Aβ variants. To this end, we carried out a systematic study of the molecular contacts described above (Phe_19_/Leu_34_, Ala_21_/Leu_34_ and Gly_25_/Ile_31_) in fibrils formed from pE_3_-Aβ_3–40_, and pE_11_-Aβ_11–40_ as well as WT-Aβ_1–40_ for comparison. This necessitates two isotopic labeling schemes, Phe_19_, Ala_21,_ and Leu_34_ (scheme I) or Gly_25_ and Ile_31_ (scheme II) for solid-state NMR spectroscopy.

**Figure 2:**
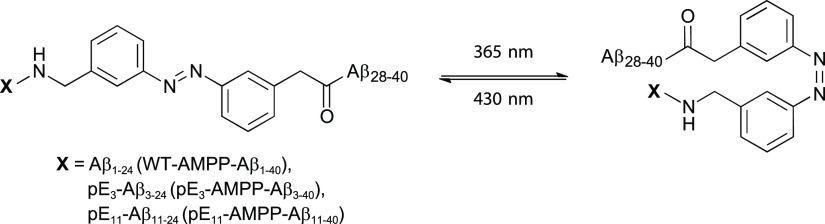
*Cis*/*trans*-conformations of the azobenzene photoswitch (3-(3-aminomethyl)phenylazo)phenylacetic acid (AMPP) introduced into Aβ_1–40_ as well as pE_3_-Aβ_3–40_ and pE_11_-Aβ_11–40_ peptides. Conformations can be switched upon irradiation with a wavelength of 365 nm (*cis*) or 430 nm (*trans*).

In an orthogonal approach, we introduced an azobenzene-based photoswitch into the unstructured linker region between the two β-strands of WT Aβ_1–40_ as well as pE_3_-Aβ_3–40_ and pE_11_-Aβ_11–40_ peptides (Figure 2). These modified peptides offer the unique possibility of forcing the peptide chain into a hairpin or an extended starting conformation. Upon irradiation with light of suitable wavelengths, the (3-(3-aminomethyl)phenylazo)phenylacetic acid (AMPP)-based photoswitch can arrange into either the *cis*- (365 nm) or the *trans*-conformation (430 nm). Simultaneously, the distinctly different UV/vis spectra of both conformations allow for an *in situ*-monitoring of the structure during fibrillation.^42,43^

## Experimental Methods

### Peptide Synthesis

Standard Fmoc solid phase synthesis was used to produce the Aβ peptides with the sequence DA**Z**FRHDSGY **Z**VHHQKLVFF AEDV**XXX**KGA IIGLMVGGVV, where (i) XXX = GSN and Z = E (WT-Aβ_1–40_) or the capping pyroglutamate (pE3-Aβ_3–40_, pE11-Aβ1_1–40_), or (ii) XXX = (3-(3-aminomethyl)phenylazo)phenylacetic acid and Z = E (WT-AMPP-Aβ_1–40_) or capping pyroglutamate (pE_3_-AMPP-Aβ_3–40_, pE_11_-AMPP-Aβ_11–40_). The modified residues are highlighted in bold in the peptide sequence. The AMPP-photoswitch was synthesized as described earlier^44^ and incorporated into the peptide by standard Fmoc solid phase synthesis. For NMR measurements, uniformly ^13^C/^15^N-labeled amino acids were introduced in the respective positions (as underlined in the peptide sequence) Phe_19_, Ala_21_ and Leu_34_ (labeling scheme I) or Gly_25_ and Ile_31_ (labeling scheme II, only for pE_11_-Aβ_11–40_ additional Leu_17_ and Glu_22_) for WT-Aβ_1–40_, pE_3_-Aβ_3–40_ and pE_11_-Aβ_11–40_. Peptide synthesis was performed by the Peptide Synthesis Core Unit of Leipzig University (https://home.uni-leipzig.de/izkf/index_peptide.html). The peptide purity level determined by HPLC analysis and MALDI mass spectrometry was ≥94% depending on the peptide (see Supporting Information).

### NMR Sample Preparation

The peptides were solubilized in 50 mM tris(hydroxymethyl)aminomethane (Tris) buffer (100 mM NaCl, 0.01% w/w NaN_3_, pH 8) at a concentration of 0.1 mg mL^–1^. For fibrillation, the peptide solutions were incubated at 37 °C and shaken at 230 rpm for 2–3 weeks. For NMR measurements, fibrils were concentrated by ultracentrifugation (ca. 200,000 × *g* for 4 h at 4 °C). The pellets were lyophilized overnight, rehydrated to 50% w/w ddH_2_O and homogenized by ten freeze–thaw cycles. Finally, the samples were transferred to 3.2 mm MAS rotors and sealed.

### Solid-State MAS NMR Spectroscopy

All MAS NMR spectra were acquired on a Bruker 600 Avance III NMR spectrometer (Bruker BioSpin GmbH, Rheinstetten, Germany) at resonance frequencies of 600.3 MHz for ^1^H and 150.96 MHz for ^13^C using a triple channel 3.2 mm MAS probe at temperature of 30 °C. The 90° pulse lengths were 4 μs for both, ^1^H and ^13^C. ^1^H–^13^C CP contact time was 1 ms at a spin lock field of ∼50 kHz, and the relaxation delay 2.5 s ^1^H dipolar decoupling during acquisition with a radio frequency field of 65 kHz was applied using Spinal64.^45^ The MAS frequency was 11,777 Hz. ^13^C chemical shifts were referenced externally, relative to TMS. ^13^C–^13^C DARR NMR spectra^46^ were acquired with a mixing time of 500 ms and 128 data points in the indirect dimension.

### Thioflavin T (ThT) and Crystal Violet (CV) Fibrillation Kinetics and Absorption Spectra

A stock solution of the peptide (1 mg of peptide dissolved in 50 μL of DMSO) was prepared. The stock solution was irradiated either at 365 nm using a 3UV-38 3UV lamp (UVP, Upland, CA, USA), or at 400–600 nm with a ∼ 450 nm emission maximum by a Philips TL 20*W*/52 SLV Medical Therapy Jaundice TL/TL-D lamp (Philips, Amsterdam, The Netherlands). Irradiation time for all experiments was 30 min. After irradiation, the stock solution was diluted with ThT- and CV-containing phosphate buffer to a final concentration of 30 μM peptide (5% v/v DMSO, 25 mM sodium phosphate, 150 mM sodium chloride, 0.01% w/v NaN_3_, pH 7.4, 20 μM ThT and 5 μM CV). Triplicates of 100 μL of this solution were transferred into a 96-well plate (Corning 96-well Half Area Black/Clear Flat Bottom Polystyrene NBS Microplate, Corning GmbH, Kaiserslautern, Germany) and sealed with optically clear film qPCR seal sheets (Labsolute, Th. Geyer GmbH & Co. KG, Renningen, Germany). The ThT fluorescence was measured with a microplate reader (Tecan Spark genomics and proteomics configuration, Tecan Group AG, Mannedorf, Switzerland) at 37 °C, using the excitation wavelength of 440 nm and emission detection at 482 nm. For CV 584 nm excitation and 620 nm emission wavelengths were used instead. Measurements were performed every 5 min directly after orbital interval shaking of 5 s. Absorption spectra were recorded in parallel with the same instrument using the same samples directly after ThT and CV measurements.

### Transmission Electron Microscopy (TEM)

A volume of 2 μL of a diluted fibril solution from the final state of ThT and CV measurements (1:20 (v/v) with ddH_2_O) was transferred onto a Formvar-film-coated copper grid. After evaporation of the solvent, the sample was stained with 1% uranyl acetate. Images were recorded on an electron microscope (Zeiss SIGMA) equipped with a STEM detector and operated with Atlas software (Zeiss NTS, Oberkochen, Germany).

## Results

### Solid-State NMR Results Suggest a Predominantly Extended Conformation of all Aβ Peptides in Fibrils

To distinguish between the two possible structural models for the Aβ peptides in mature fibrils, we conducted a series of ^13^C–^13^C DARR NMR experiments with a long mixing time (500 ms) to observe the inter-residual magnetization transfer indicating close molecular contacts (*r* < 6 Å) between interacting peptide segments. We probed three molecular contacts involving residues Phe_19_ and Leu_34_, Ala_21_ and Leu_34_ (in peptides with labeling scheme I) as well as Gly_25_ and Ile_31_ (in peptides with labeling scheme II) in mature fibrils grown from WT Aβ_1–40_, pE_3_-Aβ_3–40_, or pE_11_-Aβ_11–40_ peptides (cf. Figure 1). Preparation of a second set of NMR samples which contains a mixture of labeled and unlabeled (1:3) peptides is an effective method to differentiate between inter- and intramolecular contacts.^34,47,48^ In the isotopically diluted samples, cross peaks arising from intermolecular magnetization transfer would vanish, while the intramolecular contacts are still observable.

**Figure 3:**
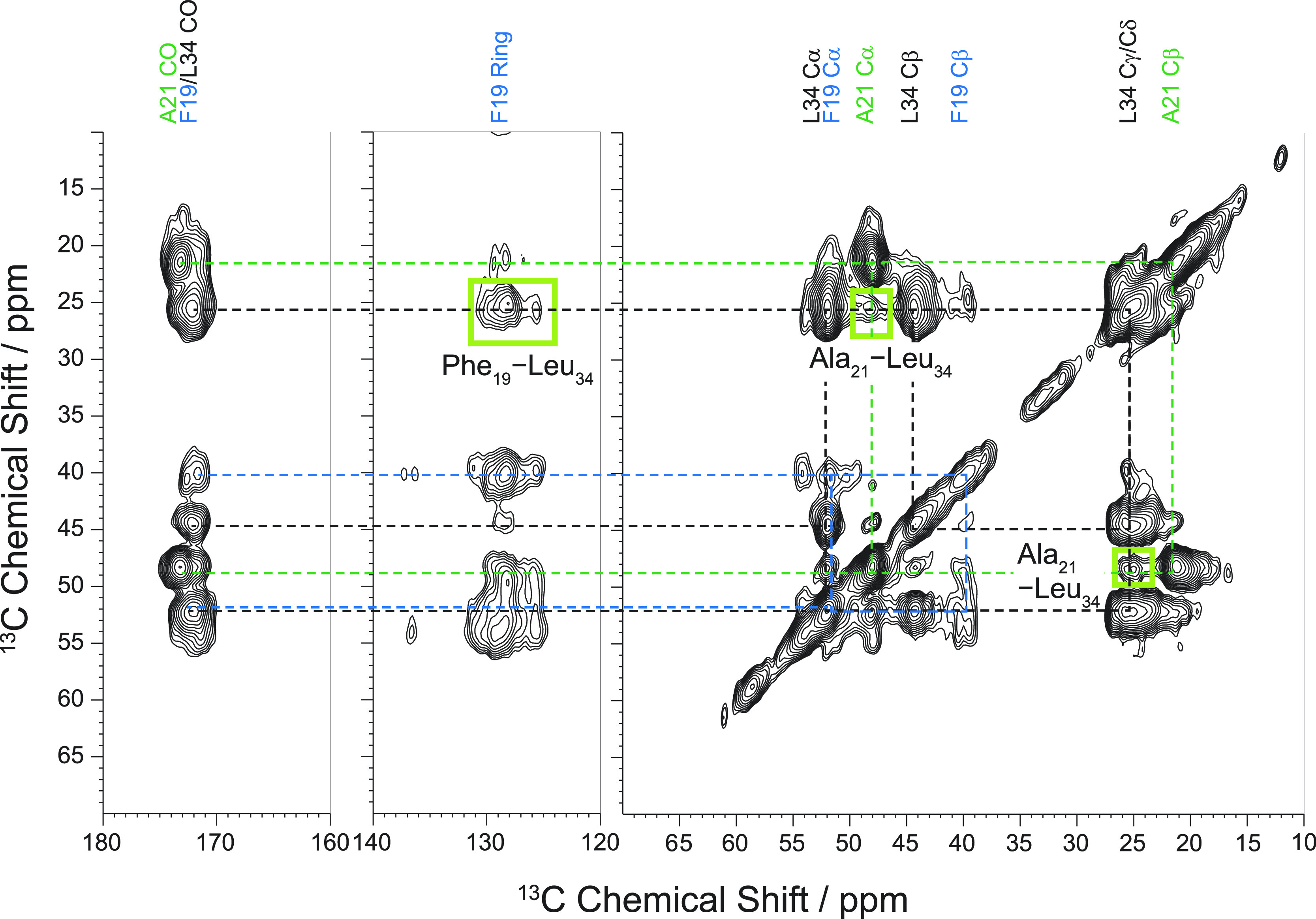
^13^C–^13^C DARR MAS NMR spectrum (500 ms mixing time) of pE_3_-Aβ_3–40_ fibrils with ^13^C/^15^N-labeled amino acids Phe_19_, Ala_21_, and Leu_34_ (labeling scheme I). Inter-residual cross peaks are highlighted by green boxes and assigned. Above the spectrum, the assignment for the diagonal peaks to the labeled amino acids is shown by following the color code: Phe_19_ – blue, Ala_21_ – green, and Leu_34_ – black. To guide the eye, intraresidual signals are connected by dashed lines.

Exemplarily, Figure 3 depicts the ^13^C–^13^C DARR MAS NMR spectrum of pE_3_-Aβ_3–40_ fibrils, where residues Phe_19_, Ala_21,_ and Leu_34_ are uniformly ^13^C/^15^N-labeled (labeling scheme I). The DARR spectra of the other fibrils are shown in Supplementary Figures S1 and S2. Inter-residual cross peaks between the side chains of Phe_19_ and Leu_34_ as well as of Ala_21_ Cα and Leu_34_ Cγ/δ are clearly observed, indicating that these residue must be close in space.

**Figure 4:**
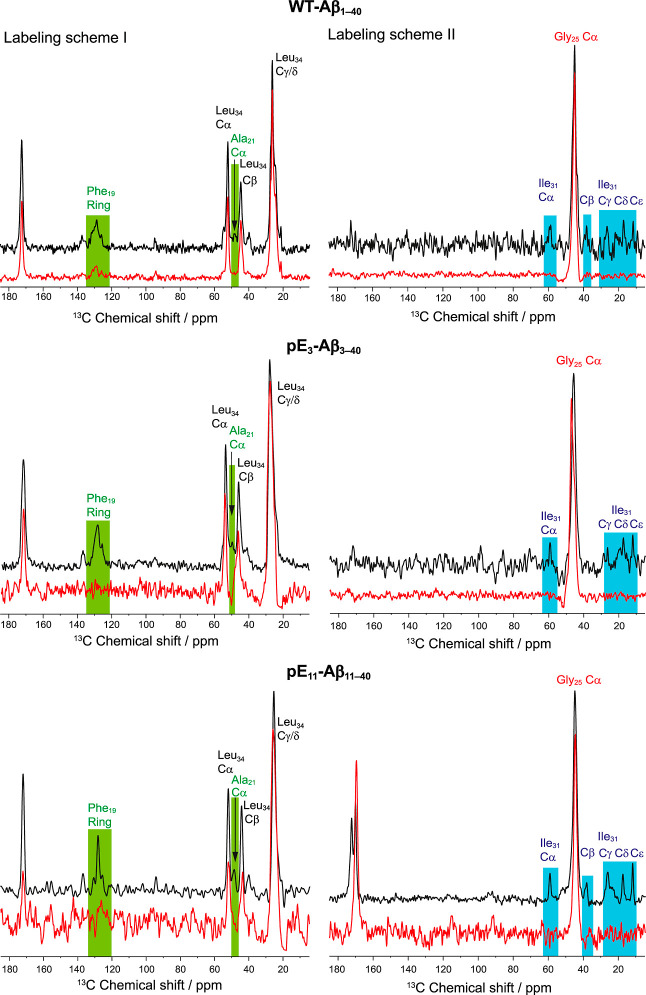
Horizontal slices from two-dimensional ^13^C–^13^C DARR MAS NMR spectra (500 ms mixing time) of investigated fibrils through the diagonal peaks of Leu_34_ Cγ/δ (labeling scheme I, left) and Gly_25_ Cα (labeling scheme II, right). The black spectra are extracted from samples containing only peptides with specifically labeled amino acids; in the red spectra, the sample contained labeled and unlabeled peptide at a molar ratio of 1:3. The signals of the investigated cross peaks are colored green or blue.

For an objective evaluation of whether molecular contacts are present in the 2D ^13^C–^13^C DARR spectra, we extracted rows (slices) from the 2D contour plots through the diagonal peak of the Leu_34_ Cγ/δ (peptides with labeling scheme I) or the Gly_25_ Cα signal (peptides with labeling scheme II) and depict them in Figure 4. The slices of the NMR spectra of fibrils of fully isotopically labeled peptides are shown in black while the spectra from the mixture of labeled and unlabeled peptides at 1:3 molar ratio are shown in red. The NMR spectra are scaled for the respective diagonal peak intensity to compare crosspeak intensity between nondiluted and diluted samples.

For fibrils grown from both pyroglutamated Aβ variants in the nondiluted samples (Figure 4, middle and bottom, black spectra) the magnetization transfer between the Phe_19_ ring (∼130 ppm) and Leu_34_ signals as well as between Ala_21_ Cα (∼48 ppm) and Leu_34_ is clearly detected as prominent cross peaks (Figure 4, left). Furthermore, in the slices through Gly_25_ Cα (Figure 4, right), cross peaks to several carbon signals of Ile_31_ are observed. Even though the signal-to-noise ratio of the NMR spectrum of pE_3_-Aβ_3–40_ fibrils (labeling scheme II) is rather poor and the signal of Ile_31_ Cβ is missing, the signals of the other four Ile_31_ carbons demonstrate the proximity between Gly_25_ and Ile_31_. All these inter-residual correlations vanish when the isotopically labeled peptides are diluted with unlabeled peptides (Figure 4, red spectra). Therefore, these contacts must be of an intermolecular nature, which is consistent with an extended conformation for the Aβ peptides in the fibrils.

For WT-Aβ_1–40_, the structural results are quite similar compared to those of the pyroglutamated Aβ variants. In the respective spectral slices (Figure 4, top), the molecular contact between Phe_19_ and Leu_34_ is manifested in the cross peak connecting the signals of the Phe_19_ ring carbons (∼130 ppm) and the signals of the side chain of Leu_34_. Also, the proximity of Leu_34_ to Ala_21_ is indicated by its corresponding cross peak (∼48 ppm). In the spectrum of the diluted sample (Figure 4, top left, red), a small residual signal for the Phe_19_–Leu_34_ Cγ/δ contact is still detectable. This may indicate that some of the monomers adopt a hairpin structure in the fibrils or that the 1:3 dilution is not sufficient for completely suppressing intramolecular contacts. Interestingly, the latest structure by the Tycko lab^33^ also shows peptides in a β-hairpin conformation in the outer cross layer in addition to the mainly extended peptides in the core of the fibril. This may explain the residual intensity in our experiments with diluted fibrils. In addition, the magnetization transfer from Gly_25_ to Ile_31_ is detected by a cross peak in the preparation of the nondiluted fibrils (Figure 4, top right). In the case of WT Aβ_1–40_, the NMR signals for the individual carbons exhibit a poor signal-to-noise ratio. Nevertheless, since all five signals of isoleucine are detected, one can be sure that there is a magnetization transfer and the low quality of the NMR spectra is caused by the small absolute amounts of labeled peptide in this sample preparation. Again, these signals vanish in the diluted samples (red spectra). Therefore, the molecular contacts between Phe_19_ and Leu_34_ as well as Gly_25_ to Ile_31_ are of intermolecular origin.

### Investigation of Aβ Peptide Variants with an Integrated Photoswitch to Predefine Them in an Extended or Hairpin Conformation Confirm the Extended Structure of Aβ_40_ Fibrils

Furthermore, we investigated what fibrillary morphologies are formed when the peptides are constrained into either a *cis*- (hairpin) or *trans*- (extended) conformation prior to the fibrillation process. To this end, the three amino acids Gly_25_, Ser_26,_ and Asn_27_ in a relatively flexible part of the fibril structures were replaced by an AMPP-photoswitch yielding AMPP-Aβ_1–40_, pE_3_-AMPP-Aβ_3–40_ and pE_11_-AMPP-Aβ_11–40_. The conformation of the AMPP-Aβ constructs can be easily monitored in real time by UV/vis spectroscopy, as the *trans*-conformer exhibits an absorption peak at ca. 330 nm, which is absent in the *cis*-conformation. In contrast, for the *cis*-conformer, a small absorption peak at 430 nm is observed (Supplementary Figure S3).^44^ Upon irradiation at ca. 450 nm (transformation into *trans*-azobenzene) or ca. 365 nm (transformation into *cis*-azobenzene), the AMPP-Aβ peptide can be transformed into the respective conformations to ensure a defined starting structure. In a dark environment and at room temperature, these conformational states are stable in aqueous solutions for several hours under the chosen experimental conditions.

**Figure 5:**
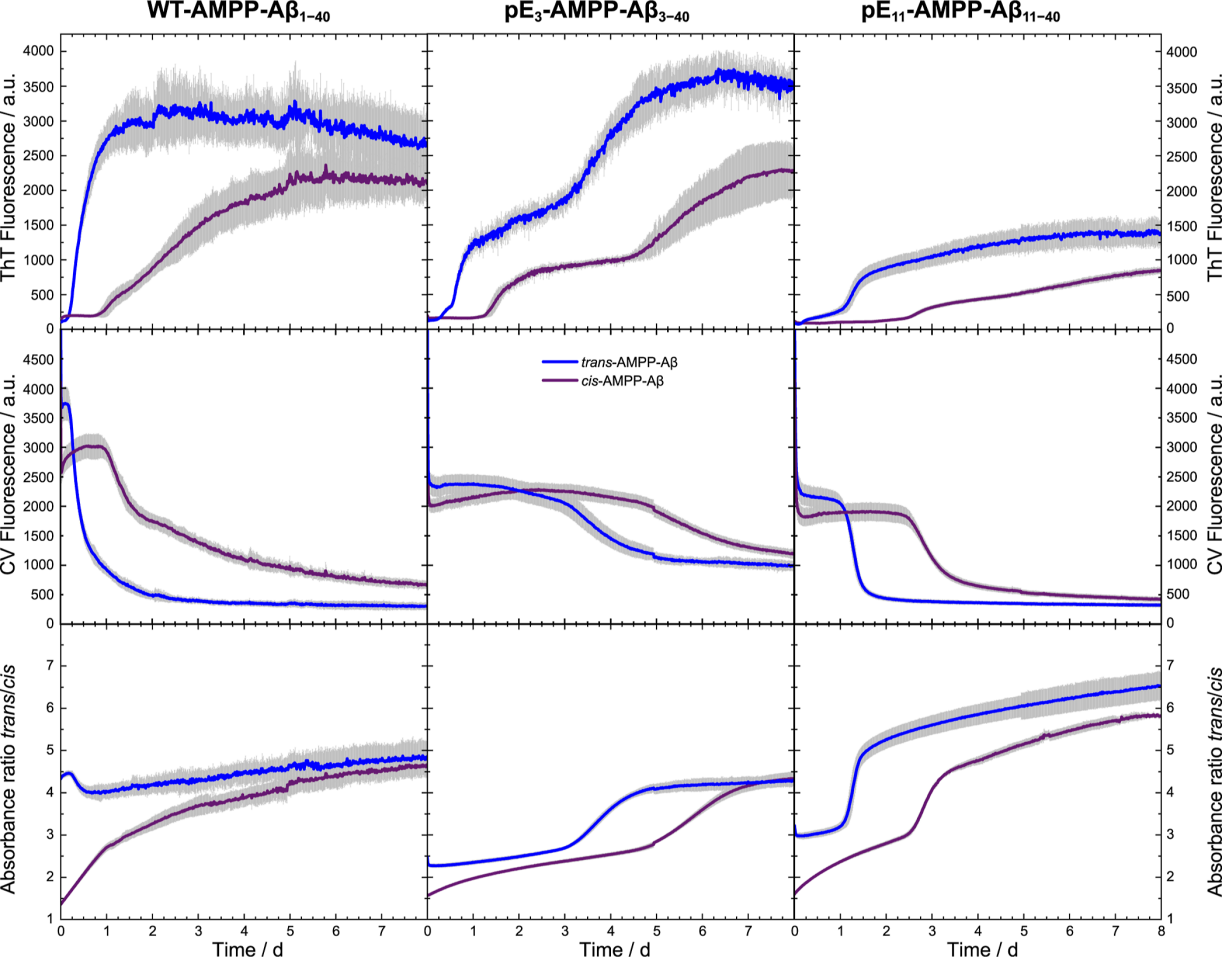
AMPP-Aβ_1–40_ (left column), pE_3_-AMPP-Aβ_3–40_ (middle column), pE_11_-AMPP-Aβ_11–40_ (right column) ThT fluorescence (top row), CV fluorescence (middle row), and AMPP UV/vis absorption (bottom row) measurements to follow the fibrillation and conformation kinetics. Sample stock solutions in DMSO were irradiated for 30 min with either blue light (ca. 450 nm, obtaining *trans*-conformation) or UV-light (365 nm, obtaining *cis*-conformation) prior to fibrillation initiation by dilution with fibrillation buffer containing ThT and CV. Three independent experiments were performed in triplicate for each measurement. The curves for initial *trans*-AMPP-Aβ conformation are plotted in blue and for the *cis*-AMPP-Aβ conformation in violet. The curves show the average values; the error bars (gray) indicate one standard deviation.

To test the influence of the two different conformational states of the AMPP-Aβ constructs on fibril formation kinetics, ThT and CV fluorescence measurements were performed. The peptide stock solution in DMSO was irradiated with either blue (ca. 450 nm) or UV light (ca. 365 nm), and fibrillation was subsequently induced by diluting the sample with aqueous fibrillation buffer. The resulting ThT and CV fibrillation kinetics for WT-AMPP-Aβ_1–40_, pE_3_-AMPP-Aβ_3–40,_ and pE_11_-AMPP-Aβ_11–40_ are shown in Figure 5 (top and middle row). At the same time, UV/vis absorption spectra were recorded, and the ratios of the absorbance at 365 nm (indicative for *trans*-AMPP) and 430 nm (indicative for *cis*-AMPP) for all peptide variants are plotted in Figure 5 (bottom row). Finally, the formation of mature fibrils could be confirmed for all samples with transmission electron microscopy (Supplementary Figure S4).

The obtained fibrillation kinetics data (ThT flourescence) show the typical three characteristic phases of amyloid formation, i.e., the lag, growth, and plateau phases, which differ for the three Aβ variants. All variants have in common that the initially *trans*-AMPP isomers have shorter lag times and shorter fibrillation times (visual estimation of ThT curves in Figure 5, data fitting not possible) than their *cis*-AMPP counterparts, otherwise, the overall shape of the curves is similar. The fibrillation curve for *trans*-AMPP-Aβ_1–40_ resembles the one for WT-Aβ_1–40_ under matching conditions confirming the WT-like behavior of AMPP-Aβ_1–40_ despite the AMPP-modification.^39^ On the other hand, pE_3_-AMPP-Aβ_3–40_ shows a biphasic ThT fibrillation as it was observed before for pE_3_-Aβ_3–40_^49^ and other Aβ variants exhibiting a phase with metastable oligomers lasting up to 2 days.^39,50,51^ The pE_11_-AMPP-Aβ_11–40_ variant fibrillates in a monophasic way, but the ThT fluorescence is significantly lower than that for both other variants. The CV fluorescence, which is sensitive for oligomeric species,^39,51^ decreases when the ThT fluorescence increases showing the transition from oligomeric species to the fibrillar state. Notably, this supports the hypothesis that the biphasic ThT fluorescence plot is indicative of a considerable proportion of oligomeric species before the second phase step (Figure 5, middle column).

Plotting the ratio of the absorption for *trans*- and *cis*-AMPP-Aβ as a function of the fibrillation time yields the curves depicted in Figure 5 (bottom row). Changes in the *cis*-/*trans*-AMPP ratio coincided timewise with changes in the ThT fluorescence plots. For instance, it seems that a ratio of *trans*- to *cis*-conformation of 2.8 to 3.3 is required for initiation of fibrillation. Also, it seems that for pE_3_-AMPP-Aβ_3–40_ and pE_11_-AMPP-Aβ_11–40_, the initial *trans*-conformation (blue curves) is not fully stable upon dilution of the DMSO stock solution into the fibrillation buffer. This, subsequently, leads to an additional increase in the *trans*-/*cis*-AMPP-Aβ ratio upon fibrillation. For the samples with initial *cis*-conformation, the final *trans*-/*cis*-AMPP-Aβ ratio is relatively similar. Interestingly, independent of which conformation represents the initial structure, the final *trans*-/*cis*-AMPP-Aβ ratio is practically the same for the respective mature fibrils, only the fibril formation time is longer for the *cis*-AMPP starting conformation.

These observations point toward a substantial amount of configurational change from *cis-* to *trans-*AMPP-Aβ prior to fibrillation, leading to the conclusion that an extended conformation is required in order to form fibrils with a final extended conformation.

## Discussion

In our previous study on the truncated and pyroglutamated mutant pE_11_-Aβ_11–40_ solid-state NMR data on mature fibrils revealed an intermolecular contact between Gly_25_ and Ile_31,_ which could not be explained by the hairpin structural models accessible at that time.^21^ In the meantime, a new structure of WT-Aβ_40_ fibrils obtained by a combination of cryo-EM and solid-state NMR has been released, which exhibits a predominantly extended conformation of the Aβ peptides.^33^ Our results for WT-Aβ_1–40_, pE_3_-Aβ_3–40_, and pE_11_-Aβ_11–40_ revealed the intermolecular nature of the three selected contact pairs Phe_19_/Leu_34_, Ala_21_/Leu_34_ as well as Gly_25_/Ile_31_ in isotopic labeling dilution experiments. The contacts detected also confirm the extended structure, as proposed by the Tycko lab,^33^ for all three Aβ fibrils samples. On the basis of these results, a hairpin structure can be excluded under the used experimental conditions. Especially, the observed Gly_25_–Ile_31_ contact is a decisive indicator for the extended structure (see Figure 1). It can be used to distinguish between the different structural models of Aβ fibrils, which are known for structural polymorphism, and the dependence of the nature of the fibrils on the fibrillation pathway.^25,26,41^

As an orthogonal method, we prepared Aβ peptides comprising a chemical modification allowing the peptide to be arrested in a conformation that would promote the hairpin (*cis*-AMPP-Aβ_1–40_) or extended structure (*trans*-AMPP-Aβ_1–40_). This was inspired by earlier studies that applied the (3-(3-aminomethyl)phenylazo)phenylacetic acid (AMPP) photoinducible switch into amyloid-like peptides^43^ and in particular Aβ_1–42_ variants.^42^ In the latter, fibrillar structures with WT-like characteristics could only be obtained with AMPP in *trans*-form; in the *cis*-form, only nonfibrillar, amorphous aggregates were formed. In our current study, for all three mutants containing the AMPP photoswitch (AMPP-Aβ_1–40_, pE_3_-AMPP-Aβ_3–40_, and pE_11_-AMPP-Aβ_11–40_), the comparison of the ThT fibrillation and the UV/vis absorption kinetics of the respective *trans*- and *cis*-AMPP-Aβ conformers indicated that in order to start fibrillation, a critical amount of *trans*-conformational peptide must be available. Starting from the *trans*-AMPP-Aβ conformer leads to faster fibrillation while starting from the *cis*-AMPP-Aβ conformer initially the *trans*-AMPP-Aβ-conformation must be formed before initiating the fibrillation stage. This was confirmed for all three Aβ variants investigated. The differences between the variants are best explained with the formation of soluble intermediate or less soluble fibrillar species (see the CV fluorescence measurements). *Trans*-AMPP-Aβ_1–40_ seems to have a relatively short lag phase quickly leading to fibrils, fibrillation starting with *cis*-AMPP-Aβ_1–40_ takes accordingly longer. The variant pE_11_-AMPP-Aβ_11–40_ behaves similarly, however, the increase of the absorption for the *trans*-conformation is considerably more pronounced than in the ThT fluorescence. It seems that both events are reciprocal to each other. Possibly, ThT does not intercalate/interact so easily with the pE_11_-AMPP-Aβ_11–40_ fibrils than the ones for the WT. The situation for pE_3_-AMPP-Aβ_3–40_ is a bit different compared with the other two variants. The biphasic ThT fibrillation curve (Figure 5) resembles metastable oligomers, which are confirmed with CV measurements.^39,50−52^

## Conclusions

Taken together, under the current experimental conditions, we can confidently assign the structure of the Aβ peptides in mature fibrils for three variants (pE_3_-Aβ_3–40_, pE_11_-Aβ_11–40_ as well as WT-Aβ_1–40_) to the extended structure. Especially, the molecular contact Gly_25_–Ile_31_ observed by solid-state NMR can be used to distinguish between the extended and the hairpin model of Aβ_40_.

Unlike the results of Doran and co-workers for Aβ_42_,^42^ we obtained fibrils with Aβ peptides for both the starting conformations as proven by TEM imaging (Supplementary Figure S4). Also, we established a method to simultaneously monitor the fibril- and oligomer-formation process via ThT and CV fluorescence measurements as well as the conformation (hairpin or extended) of the peptides by UV/vis measurements, which is a unique way to monitor the dynamic and complex structure forming process during fibrillation with three different probes.

## Supporting Information Available

2D DARR NMR spectra for all Aβ variants, absorption spectra for all AMPP variants at different time points, TEM images for all AMPP variants, analytical HPLC measurements, and MALDI mass spectra for all used synthetic peptides (PDF)
