# Supplementary material for: Conformation of Pyroglutamated Amyloid β (3–40) and (11–40) Fibrils – Extended or Hairpin?
Source: J Phys Chem B. 2024 Feb 9;128(7):1647–55. doi: 10.1021/acs.jpcb.3c07285 (PMC10895672; doi:10.1021/acs.jpcb.3c07285)
Supplement: Supplementary file 1 — jp3c07285_si_001.pdf [file jp3c07285_si_001.pdf]

Supplementary Information

The Conformation of Pyroglutamated Amyloid β (3–40) and (11–40) Fibrils – Extended or Hairpin?

Holger A. Scheidt[1,*], Alexander Korn[1], Benedikt Schwarze[1], Martin Krueger[2], Daniel Huster[1]

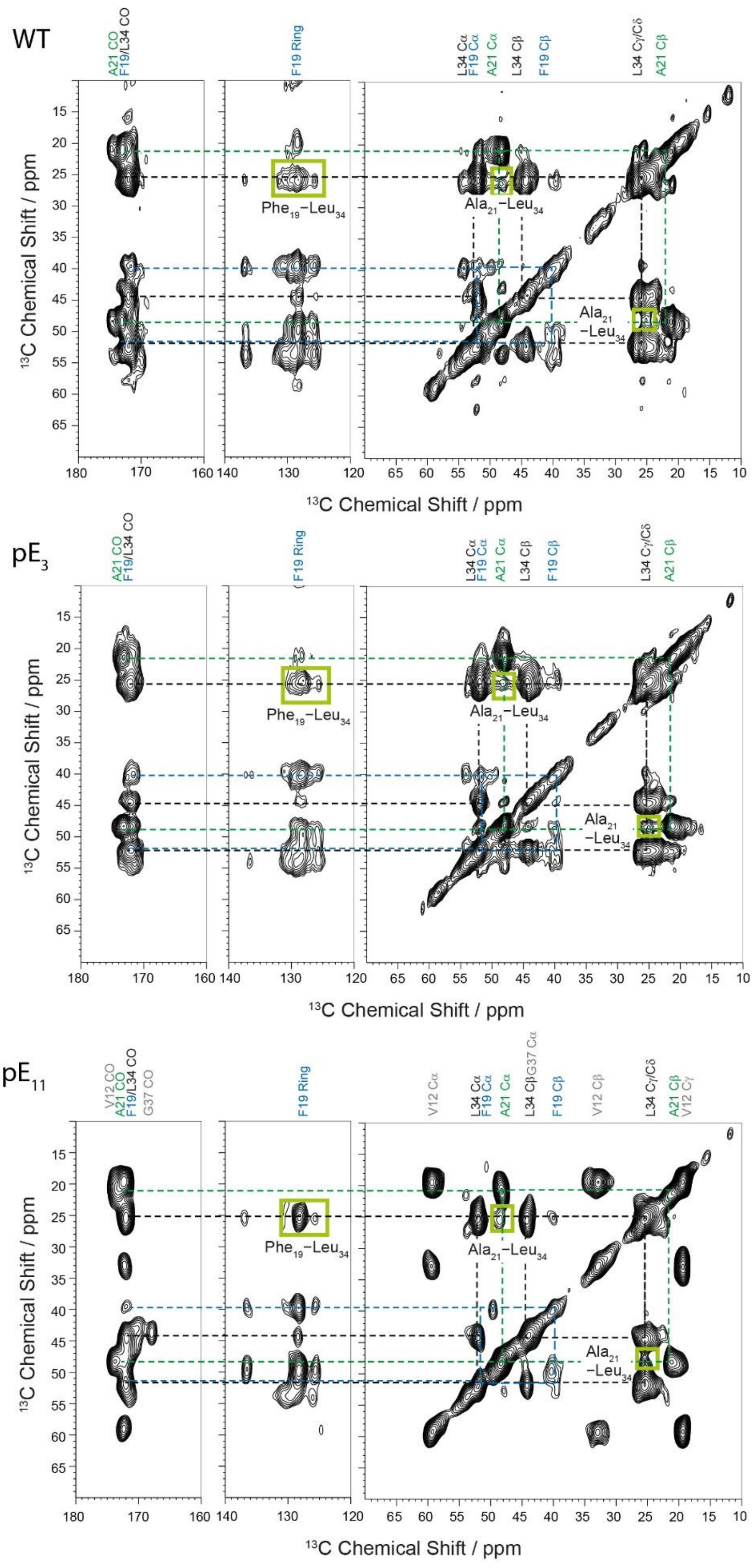

Supplementary Figure S1. ^{13}C-^{13}C DARR MAS NMR spectrum (500 ms mixing time) of all WT-A$\beta_{1\text{-}40}$ (above), pE$_{3}$-A$\beta_{3\text{-}40}$ (middle) and pE$_{11}$-A$\beta_{11\text{-}40}$ fibrils with ^{13}C/^{15}N-labeled amino acids Phe$_{19}$, Ala$_{21}$ and Leu$_{34}$ (labeling scheme I). Inter-residual cross peaks are highlighted by green boxes and assigned. Above the spectrum, the assignment for the diagonal peaks to the labeled amino acids is shown following the color code: Phe$_{19}$ – blue, Ala$_{21}$ – green, Leu$_{34}$ – black. To guide the eye, intra-residual signals are connected by dashed lines.

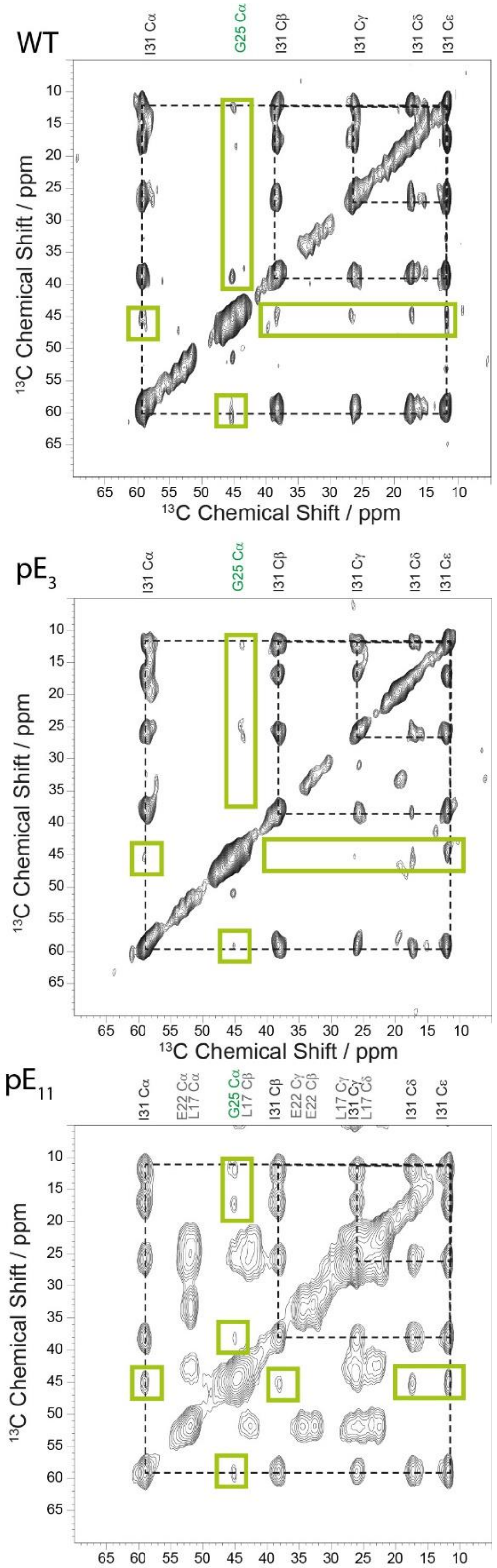

Supplementary Figure S2. ^{13}C-^{13}C DARR MAS NMR spectrum (500 ms mixing time) of WT-Aβ$_{1-40}$ (above), pE$_3$-Aβ$_{3-40}$ (middle) and pE$_{11}$-Aβ$_{11-40}$ fibrils with ^{13}C/^{15}N-labeled amino acids Gly$_{25}$ and Ile$_{31}$ (labeling scheme II). Inter-residual cross peaks are highlighted by green boxes and assigned. Above the spectrum, the assignment for the diagonal peaks to the labeled amino acids is shown. To guide the eye, intra-residual signals are connected by dashed lines.

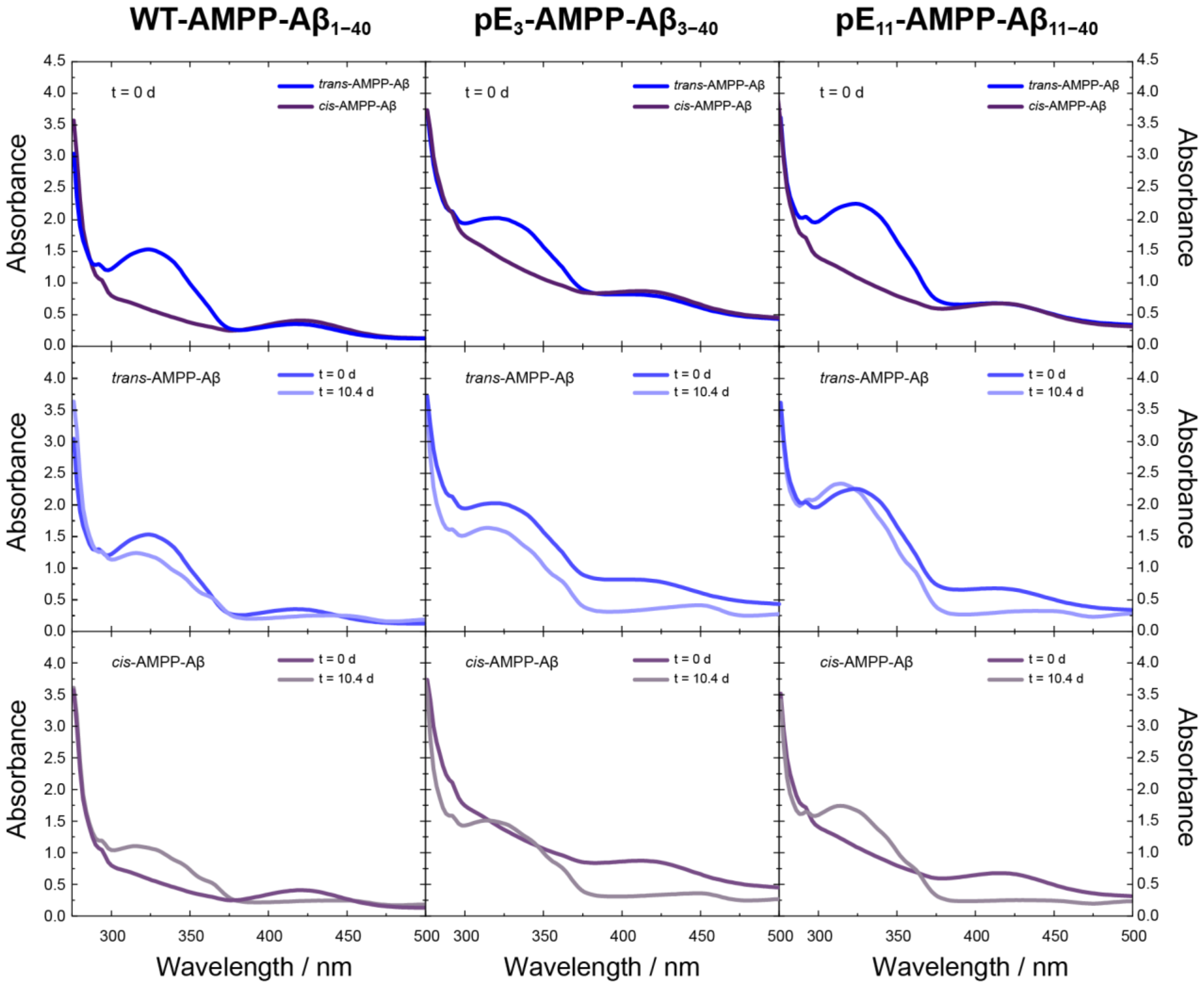

Supplementary Figure S3: Absorption spectra of **WT-AMPP-A$\beta_{1\text{–}40}$**, **pE$_3$-AMPP-A$\beta_{3\text{–}40}$** and **pE$_{11}$-AMPP-A$\beta_{11\text{–}40}$**. The upper row compares the starting points at t = 0 d for *trans*-/*cis*-AMPP-Aβ verifying the different starting structures. The middle row represents the absorption spectra for *trans*-AMPP-Aβ at t = 0 d and t = 10.4 d showing the large *trans*-AMPP proportion before and after fibrillation. The bottom row depicts the absorption spectra for *cis*-AMPP-Aβ at t = 0 d and t = 10.4 d showing the conformation change from *cis*- to *trans*-AMPP upon fibrillation.

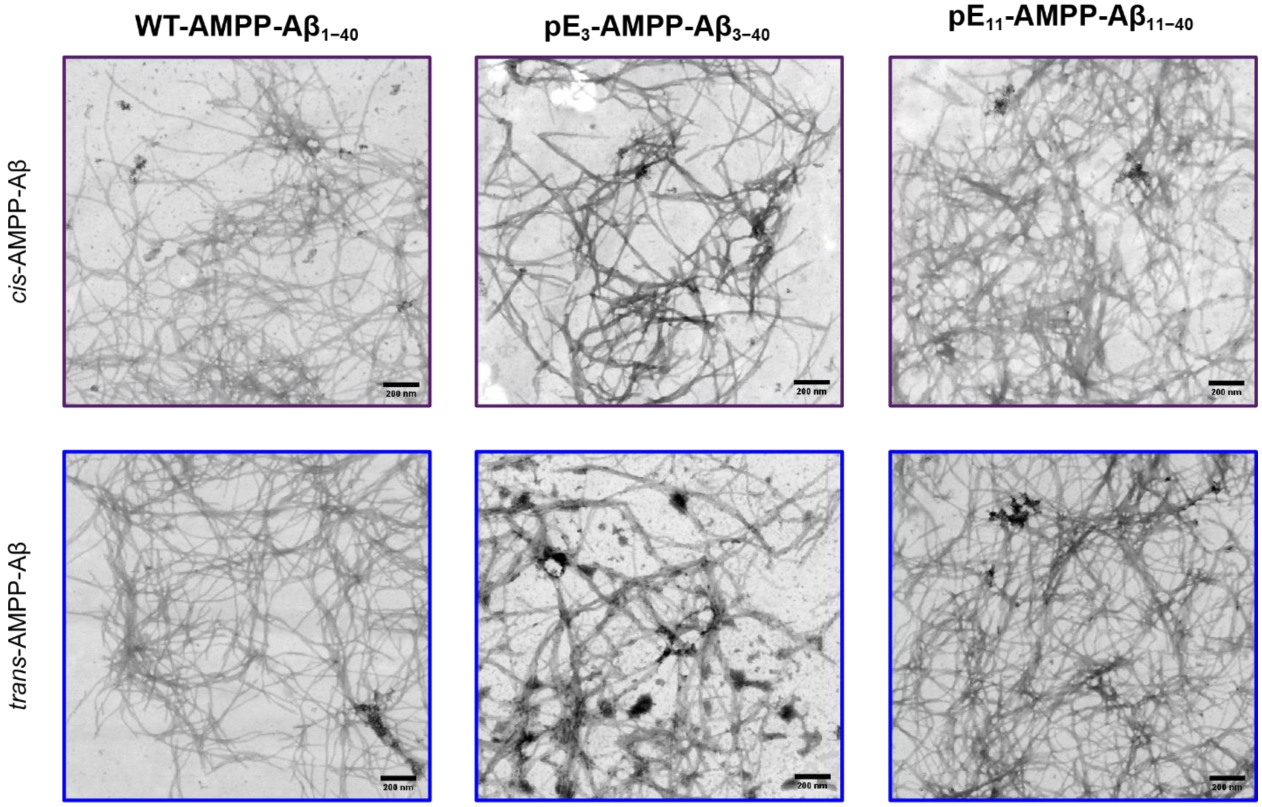

Supplementary Figure S4: TEM images of all the AMPP-incorporating variants which fibrillated during ThT and CV fluorescence assay. The upper panel represents the fibrils formed from the initial *cis*-AMPP-Aβ conformers (irradiated with UV light, violet frame), the bottom panel shows the fibrils formed from the initial *trans*-AMPP-Aβ conformers (irradiated with blue light, blue frame). The scale bar represents 200 nm.

Table S1. Assignment of sample names in chromatograms from analytical HPLC measurements and MALDI mass spectra to numbering in this manuscript.

Sample name	Numbering
AX21_purified	WT-A$\beta_{1–40}$ (^{13}C/^{15}N-labeled, scheme I)
AW21_purified	WT-A$\beta_{1–40}$ (^{13}C/^{15}N-labeled, scheme II)
X21_purified	pE$_3$-A$\beta_{3–40}$ (^{13}C/^{15}N-labeled, scheme I)
Y21_purified	pE$_3$-A$\beta_{3–40}$ (^{13}C/^{15}N-labeled, scheme II)
V21_purified	pE$_{11}$-A$\beta_{11–40}$ (^{13}C/^{15}N-labeled, scheme I)
ET16_purified	pE$_{11}$-A$\beta_{11–40}$ (^{13}C/^{15}N-labeled, scheme II)
AD23_purified	WT-AMPP-A$\beta_{1–40}$
AE23_purified	pE$_3$-AMPP-A$\beta_{3–40}$
AF23_purified	pE$_{11}$-AMPP-A$\beta_{11–40}$

214 **IZKF Leipzig, Core Unit Peptid-Technologien**
Liebigstraße 21, 04103 Leipzig, e-mail: sven_r@yahoo.com, Tel.: 0341 - 9715898 / 897

Comment:

Sample Name:	**AX21_purified**	*Injection Volume:*	**20.0**
Vial Number:	**BE2**	*Channel:*	**UV_VIS_1**
Sample Type:	**unknown**	*Wavelength:*	**220.0**
Control Program:	**Peptide_3D_basic_short**	*Bandwidth:*	**4**
Quantif. Method:	**peptide_izkf**	*Dilution Factor:*	**1.0000**
Recording Time:	**14/5/2021 10:54**	*Operator:*	**KEYUSER**
Sample ID:		*Sample Amount:*	**1.0000**

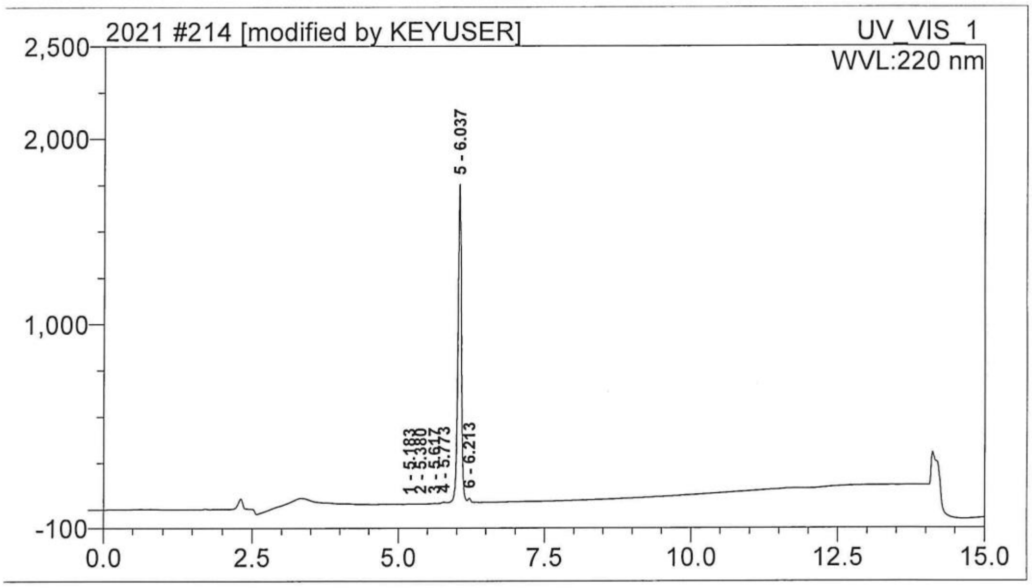

No.	Ret.Time min	Peak Name	Height mAU	Rel.Area %	Area mAU*min	Amount	Type
1	5.18	n.a.	0.8	0.07	0.09	n.a.	BM
2	5.38	n.a.	0.8	0.09	0.11	n.a.	MB
3	5.62	n.a.	0.8	0.05	0.06	n.a.	BMB
4	5.77	n.a.	6.3	0.48	0.57	n.a.	BM
5	6.04	n.a.	1717.4	98.14	117.81	n.a.	M
6	6.21	n.a.	24.9	1.17	1.40	n.a.	MB
**Total:**			1751.035	100.000	120.05	0.000	

Dr. Sven Rothemund, IZKF Leipzig, Liebigstrasse 21, 04103 Leipzig, Tel.: 0341-9715898, sven_r@yahoo.com

Comment 1	AX21_purified
Comment 2	MW:4350,9(M+H)+

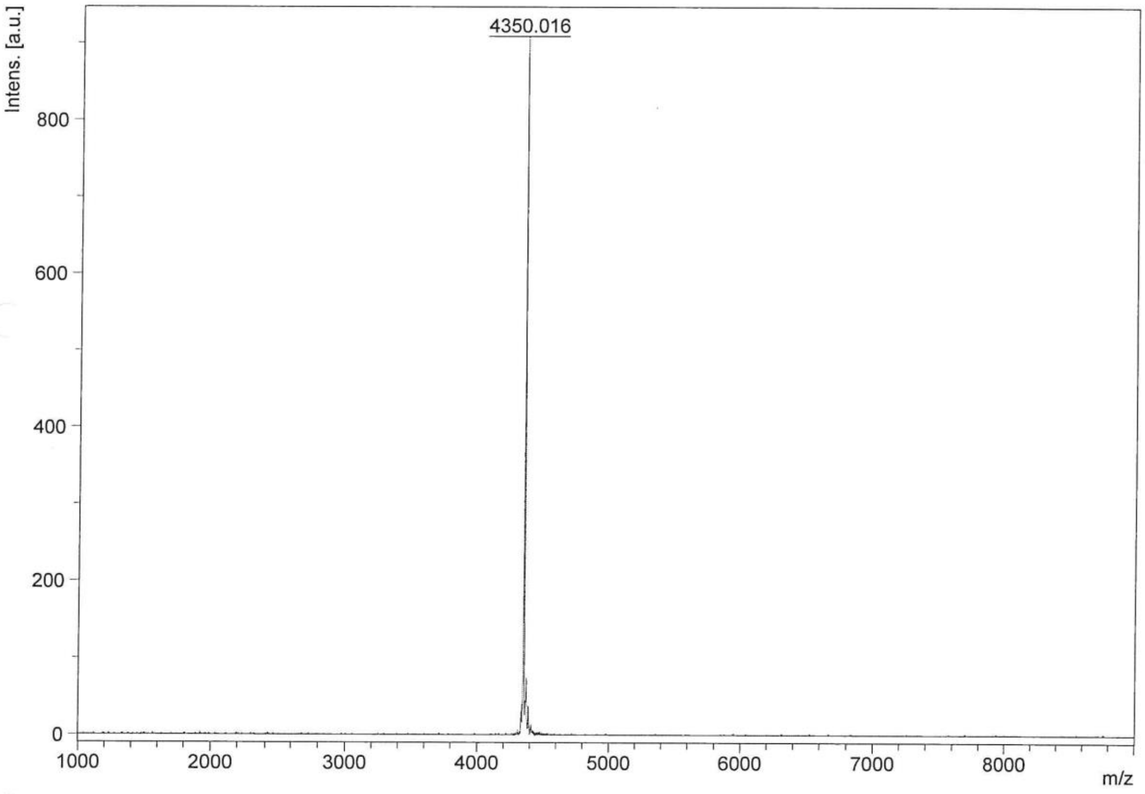

Acquisition Parameter

Date of acquisition	2021-05-14T10:27:15.540+02:00
Acquisition method name	D:\Methods\flexControlMethods\LP_PepMix.par
Aquisition operation mode	Linear
Voltage polarity	POS
Number of shots	100
Name of spectrum used for calibration	
Calibration reference list used	PeptideCalibStandardInsulin2 monoAv

Instrument Info

User	IZKF
Instrument	FLEX-PC

D:\Data\IZKF2021\AX21_purified\0_G3\2 printed: 14.05.2021 10:28:40

210 IZKF Leipzig, Core Unit Peptid-Technologien
Liebigstraße 21, 04103 Leipzig, e-mail: sven_r@yahoo.com, Tel.: 0341 - 9715898 / 897

Comment:

Sample Name:	AW21_purified	*Injection Volume:*	20.0
Vial Number:	BB1	*Channel:*	UV_VIS_1
Sample Type:	unknown	*Wavelength:*	220.0
Control Program:	Peptide_3D_basic_short	*Bandwidth:*	4
Quantif. Method:	peptide_izkf	*Dilution Factor:*	1.0000
Recording Time:	12/5/2021 12:42	*Operator:*	KEYUSER
Sample ID:		*Sample Amount:*	1.0000

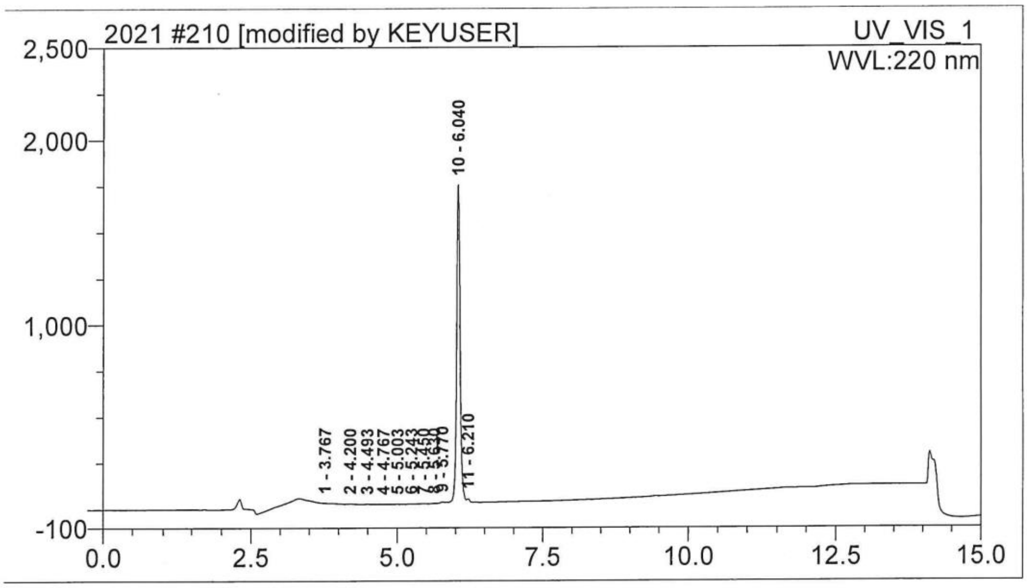

No.	Ret.Time min	Peak Name	Height mAU	Rel.Area %	Area mAU*min	Amount	Type
1	3.77	n.a.	0.0	0.16	0.19	n.a.	BMB
2	4.20	n.a.	1.2	0.14	0.17	n.a.	BMB
3	4.49	n.a.	1.1	0.12	0.14	n.a.	BMB
4	4.77	n.a.	0.8	0.08	0.09	n.a.	BMB
5	5.00	n.a.	0.9	0.08	0.10	n.a.	BMB
6	5.24	n.a.	0.9	0.09	0.11	n.a.	BMB
7	5.45	n.a.	0.9	0.09	0.11	n.a.	BMB
8	5.63	n.a.	0.9	0.06	0.08	n.a.	BMb*
9	5.77	n.a.	6.7	0.52	0.62	n.a.	bM
10	6.04	n.a.	1722.1	97.66	116.25	n.a.	M
11	6.21	n.a.	21.7	0.99	1.18	n.a.	MB
Total:			1757.211	100.000	119.03	0.000	

Chromeleon (c) Dionex 1996-2006
Version 6.80 SR15 Build 4656 (243203)

Dr. Sven Rothemund, IZKF Leipzig, Liebigstrasse 21, 04103 Leipzig, Tel.: 0341-9715898, sven_r@yahoo.com

Comment 1	AW21_purified
Comment 2	MW:4339,9(M+H)+

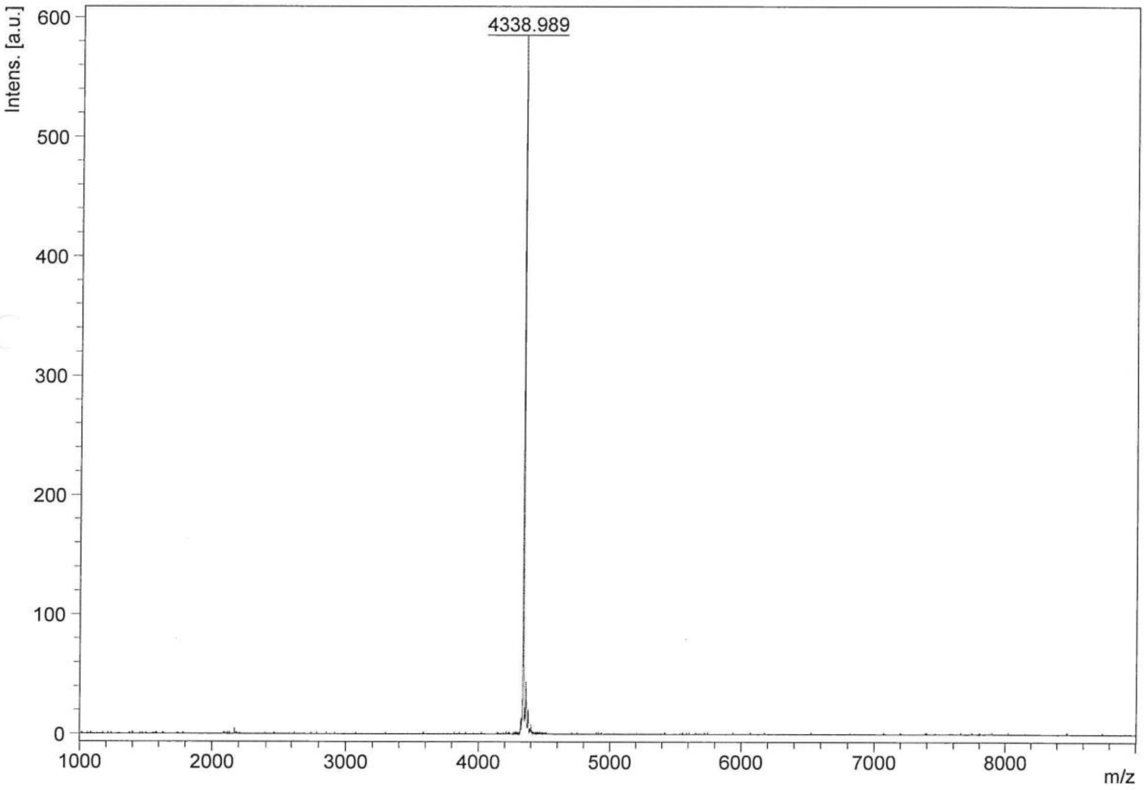

Acquisition Parameter

Date of acquisition	2021-05-12T12:02:53.555+02:00
Acquisition method name	D:\Methods\flexControlMethods\LP_PepMix.par
Aquisition operation mode	Linear
Voltage polarity	POS
Number of shots	100
Name of spectrum used for calibration	
Calibration reference list used	PeptideCalibStandardInsulin2 monoAv

Instrument Info

User	IZKF
Instrument	FLEX-PC

D:\Data\IZKF2021\AW21_purified\0_E4\1 printed: 12.05.2021 12:05:00

113 IZKF Leipzig, Core Unit Peptid-Technologien
Liebigstraße 21, 04103 Leipzig, e-mail: sven_r@yahoo.com, Tel.: 0341 - 9715898 / 897

Comment:

Sample Name:	**X21_purified**	*Injection Volume:*	**20.0**
Vial Number:	**RC1**	*Channel:*	**UV_VIS_1**
Sample Type:	**unknown**	*Wavelength:*	**220.0**
Control Program:	**Peptide_3D_basic_short**	*Bandwidth:*	**4**
Quantif. Method:	**peptide_izkf**	*Dilution Factor:*	**1.0000**
Recording Time:	**17/3/2021 11:50**	*Operator:*	**KEYUSER**
Sample ID:		*Sample Amount:*	**1.0000**

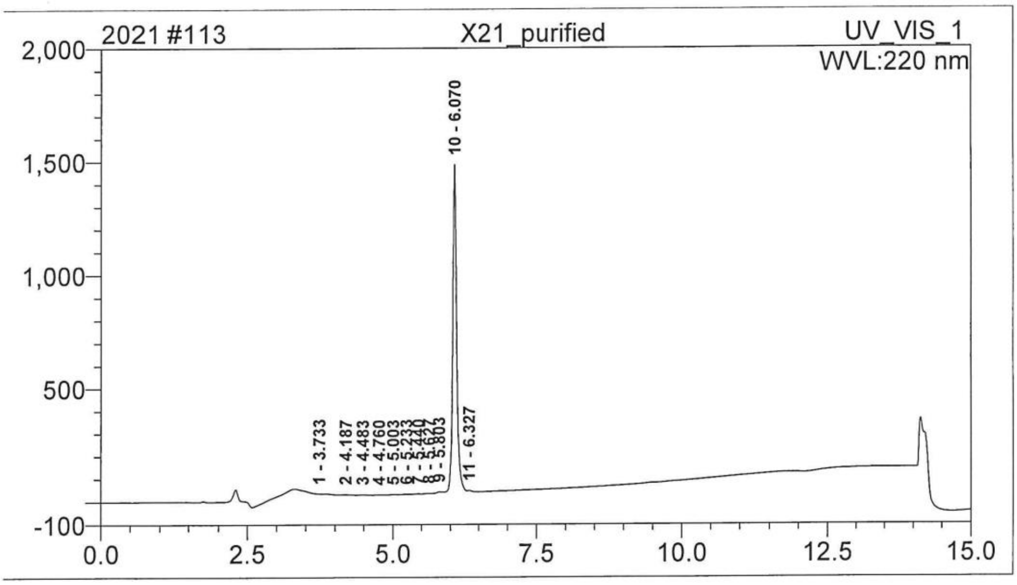

No.	Ret.Time min	Peak Name	Height mAU	Rel.Area %	Area mAU*min	Amount	Type
1	3.73	n.a.	0.0	0.19	0.22	n.a.	BMB
2	4.19	n.a.	1.2	0.15	0.17	n.a.	BMB
3	4.48	n.a.	1.0	0.10	0.12	n.a.	BMB
4	4.76	n.a.	0.7	0.07	0.08	n.a.	BMB
5	5.00	n.a.	0.7	0.07	0.08	n.a.	BMB
6	5.23	n.a.	0.9	0.09	0.10	n.a.	BMB
7	5.44	n.a.	1.1	0.09	0.10	n.a.	BMB
8	5.63	n.a.	0.9	0.09	0.11	n.a.	BMB
9	5.80	n.a.	5.7	0.50	0.60	n.a.	bM
10	6.07	n.a.	1447.1	98.07	116.54	n.a.	M
11	6.33	n.a.	7.7	0.59	0.70	n.a.	MB
**Total:**			1467.123	100.000	118.83	0.000	

Chromeleon (c) Dionex 1996-2006
Version 6.80 SR15 Build 4656 (243203)

Dr. Sven Rothemund, IZKF Leipzig, Liebigstrasse 21, 04103 Leipzig, Tel.: 0341-9715898, sven_r@yahoo.com

Comment 1 X21_purified
Comment 2 MW:4145,8(M+H)+

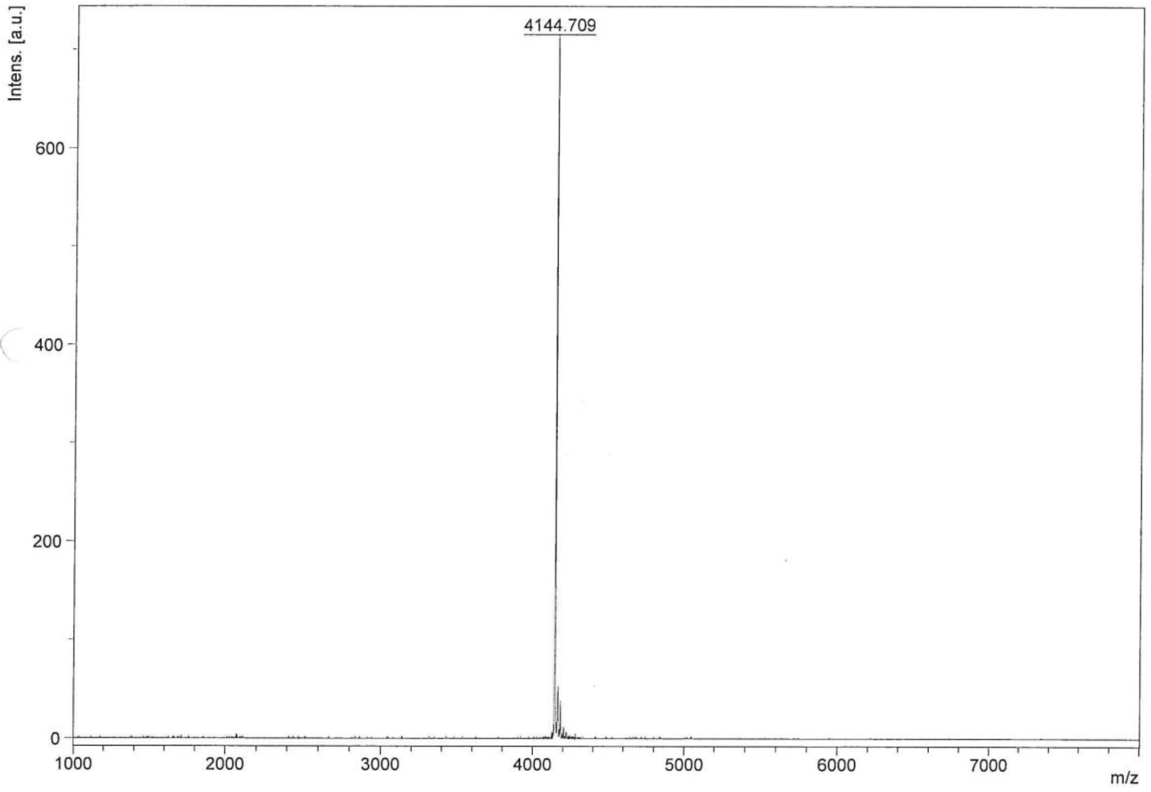

Acquisition Parameter

Date of acquisition	2021-03-17T11:58:06.749+01:00
Acquisition method name	D:\Methods\flexControlMethods\LP_PepMix.par
Aquisition operation mode	Linear
Voltage polarity	POS
Number of shots	71
Name of spectrum used for calibration	
Calibration reference list used	PeptideCalibStandardInsulin2 monoAv

Instrument Info

User	IZKF
Instrument	FLEX-PC

D:\Data\IZKF2021\X21_purified\0_F11\1 printed: 17.03.2021 12:00:16

117 IZKF Leipzig, Core Unit Peptid-Technologien
Liebigstraße 21, 04103 Leipzig, e-mail: sven_r@yahoo.com, Tel.: 0341 - 9715898 / 897

Comment:

Sample Name:	**Y21_purified**	*Injection Volume:*	**20.0**
Vial Number:	**BC2**	*Channel:*	**UV_VIS_1**
Sample Type:	**unknown**	*Wavelength:*	**220.0**
Control Program:	**Peptide_3D_basic_short**	*Bandwidth:*	**4**
Quantif. Method:	**peptide_izkf**	*Dilution Factor:*	**1.0000**
Recording Time:	**23/3/2021 11:42**	*Operator:*	**KEYUSER**
Sample ID:		*Sample Amount:*	**1.0000**

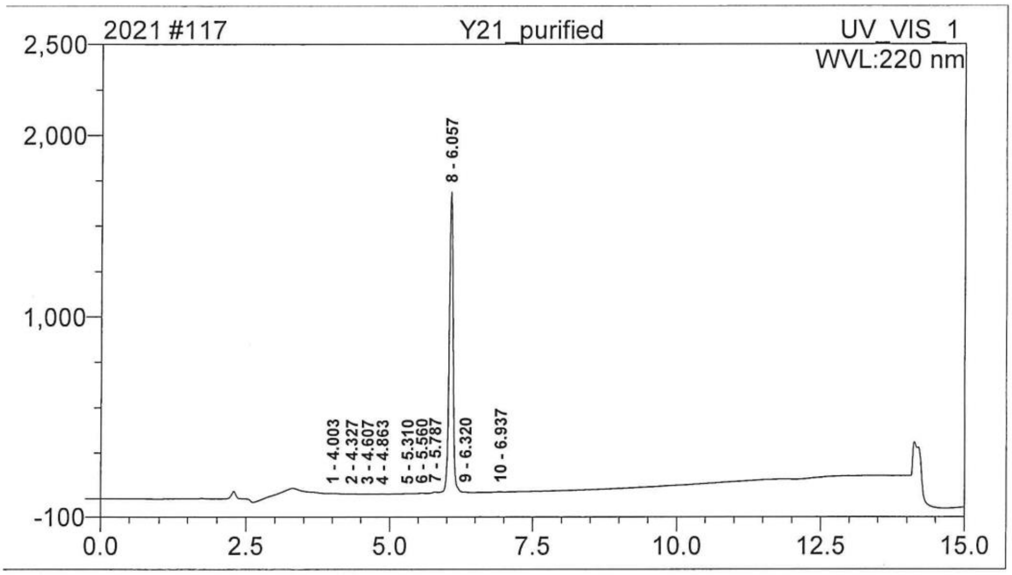

No.	Ret.Time min	Peak Name	Height mAU	Rel.Area %	Area mAU*min	Amount	Type
1	4.00	n.a.	1.4	0.16	0.21	n.a.	BMB
2	4.33	n.a.	1.1	0.12	0.15	n.a.	BMB
3	4.61	n.a.	0.8	0.07	0.10	n.a.	BMB
4	4.86	n.a.	0.8	0.07	0.10	n.a.	BMB
5	5.31	n.a.	0.6	0.06	0.08	n.a.	BMB
6	5.56	n.a.	2.5	0.19	0.25	n.a.	BM
7	5.79	n.a.	7.2	0.54	0.71	n.a.	M
8	6.06	n.a.	1656.8	98.45	130.74	n.a.	M
9	6.32	n.a.	4.1	0.25	0.34	n.a.	M
10	6.94	n.a.	0.8	0.09	0.12	n.a.	MB
**Total:**			1676.141	100.000	132.79	0.000	

Dr. Sven Rothemund, IZKF Leipzig, Liebigstrasse 21, 04103 Leipzig, Tel.: 0341-9715898, sven_r@yahoo.com

Comment 1	Y21_purified
Comment 2	MW:4134,8(M+H)+

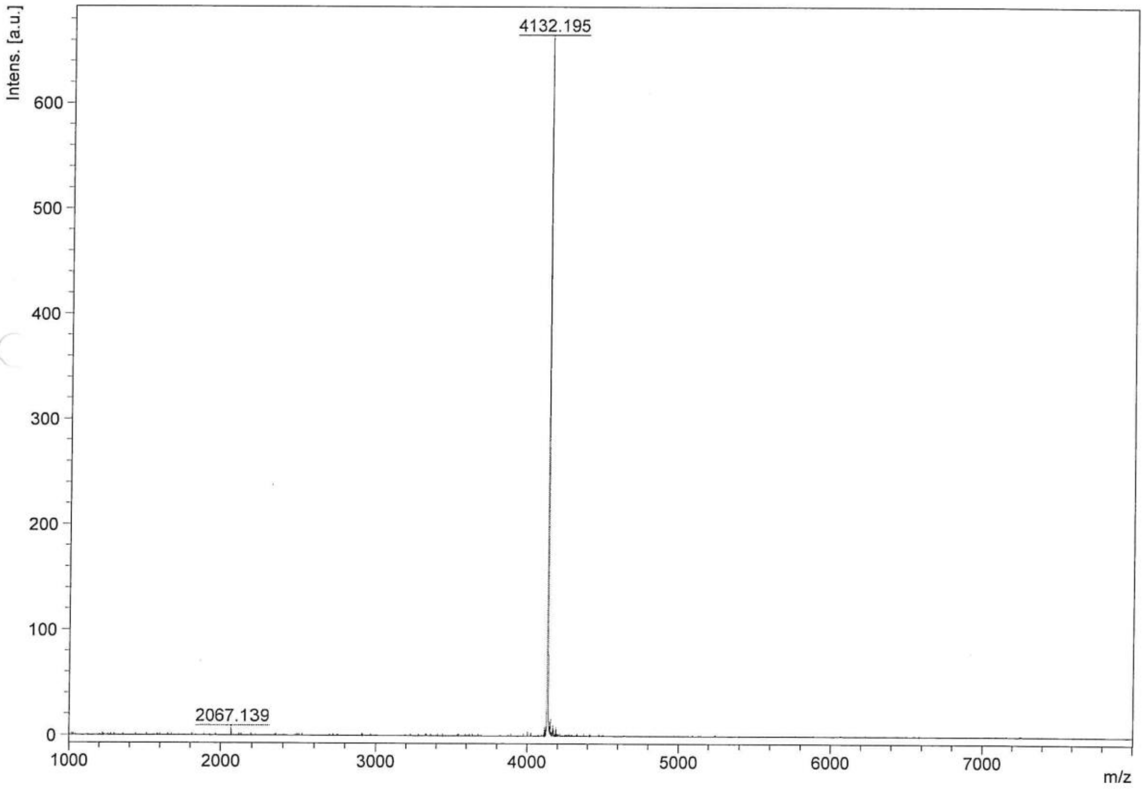

Acquisition Parameter

Date of acquisition	2021-03-23T11:43:16.001+01:00
Acquisition method name	D:\Methods\flexControlMethods\LP_PepMix.par
Aquisition operation mode	Linear
Voltage polarity	POS
Number of shots	100
Name of spectrum used for calibration	
Calibration reference list used	PeptideCalibStandardInsulin2 monoAv

Instrument Info

User	IZKF
Instrument	FLEX-PC

D:\Data\IZKF2021\Y21_purified\0_C4\1 printed: 23.03.2021 11:44:47

112 IZKF Leipzig, Core Unit Peptid-Technologien
Liebigstraße 21, 04103 Leipzig, e-mail: sven_r@yahoo.com, Tel.: 0341 - 9715898 / 897

Comment:

Sample Name:	**V21_purified**	*Injection Volume:*	**20.0**
Vial Number:	**RE1**	*Channel:*	**UV_VIS_1**
Sample Type:	**unknown**	*Wavelength:*	**220.0**
Control Program:	**Peptide_3D_basic_short**	*Bandwidth:*	**4**
Quantif. Method:	**peptide_izkf**	*Dilution Factor:*	**1.0000**
Recording Time:	**17/3/2021 14:29**	*Operator:*	**KEYUSER**
Sample ID:		*Sample Amount:*	**1.0000**

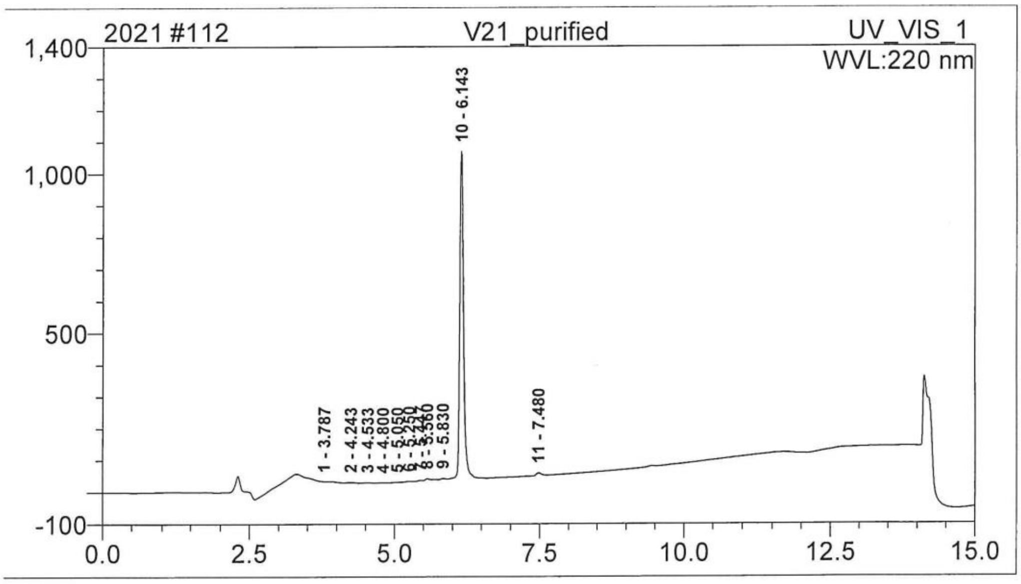

No.	Ret.Time min	Peak Name	Height mAU	Rel.Area %	Area mAU*min	Amount	Type
1	3.79	n.a.	0.0	0.26	0.21	n.a.	BMB
2	4.24	n.a.	1.2	0.22	0.17	n.a.	BMB
3	4.53	n.a.	0.9	0.15	0.12	n.a.	BMB
4	4.80	n.a.	0.7	0.11	0.08	n.a.	BMB
5	5.05	n.a.	0.7	0.09	0.08	n.a.	BMB
6	5.25	n.a.	1.5	0.21	0.16	n.a.	BMB
7	5.45	n.a.	1.2	0.13	0.10	n.a.	BMB
8	5.56	n.a.	5.2	0.57	0.45	n.a.	BMB
9	5.83	n.a.	3.4	0.36	0.29	n.a.	bM
10	6.14	n.a.	1028.0	96.84	77.47	n.a.	MB
11	7.48	n.a.	9.0	1.06	0.85	n.a.	BMB
**Total:**			1051.751	100.000	80.00	0.000	

Comment 1	V21_purified
Comment 2	MW:3153,8(M+H)+

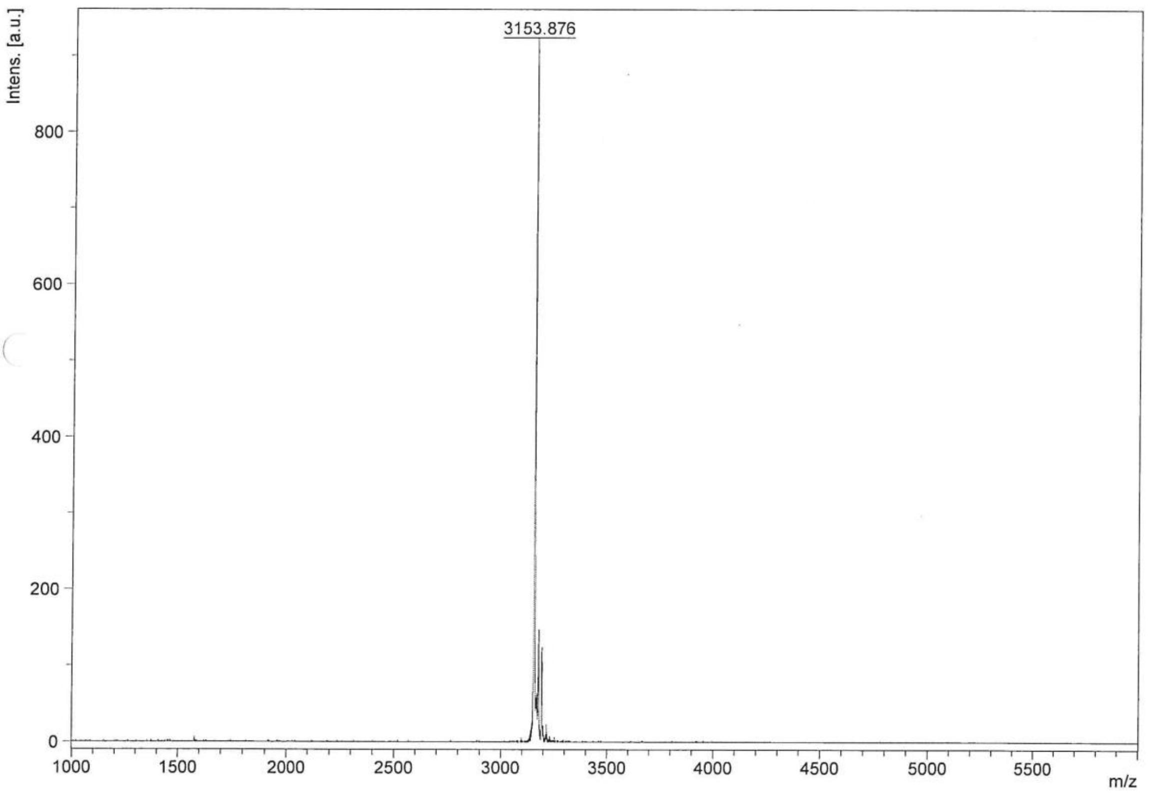

Acquisition Parameter

Date of acquisition	2021-03-17T13:47:29.198+01:00
Acquisition method name	D:\Methods\flexControlMethods\LP_PepMix.par
Aquisition operation mode	Linear
Voltage polarity	POS
Number of shots	75
Name of spectrum used for calibration	
Calibration reference list used	PeptideCalibStandardInsulin2 monoAv

Instrument Info

User	IZKF
Instrument	FLEX-PC

D:\Data\IZKF2021\V21_purified\0_G10\1 printed: 17.03.2021 13:51:25

9 IZKF Leipzig, Core Unit Peptid-Technologien

Liebigstraße 21, 04103 Leipzig, e-mail: sven_r@yahoo.com, Tel.: 0341 - 9715898 / 897

Comment:

Sample Name:	**ET16_purified**	*Injection Volume:*	**20,0**
Vial Number:	**GE6**	*Channel:*	**UV_VIS_1**
Sample Type:	**unknown**	*Wavelength:*	**220.0**
Control Program:	**Peptide_3D_basic_short**	*Bandwidth:*	**4**
Quantif. Method:	**peptide_izkf**	*Dilution Factor:*	**1,0000**
Recording Time:	**4.1.2017 12:53**	*Operator:*	**KEYUSER**
Sample ID:		*Sample Amount:*	**1,0000**

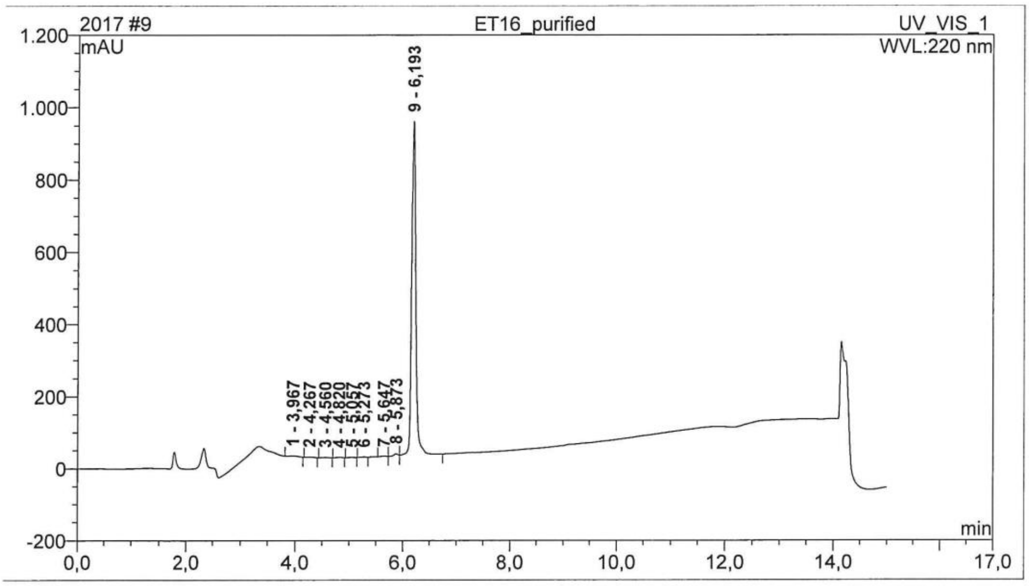

No.	Ret.Time min	Peak Name	Height mAU	Rel.Area %	Area mAU*min	Amount	Type
1	3,97	n.a.	2,0	0,38	0,32	n.a.	BMB
2	4,27	n.a.	0,8	0,13	0,11	n.a.	BMB
3	4,56	n.a.	0,9	0,14	0,12	n.a.	BMB
4	4,82	n.a.	0,7	0,09	0,08	n.a.	BMB
5	5,06	n.a.	0,5	0,06	0,05	n.a.	BMB
6	5,27	n.a.	0,6	0,08	0,07	n.a.	BMB
7	5,65	n.a.	1,1	0,14	0,12	n.a.	BM
8	5,87	n.a.	6,2	0,64	0,54	n.a.	M
9	6,19	n.a.	924,5	98,34	83,15	n.a.	MB
**Total:**			937,343	100,000	84,56	0,000	

Dr. Sven Rothemund, IZKF Leipzig, Liebigstrasse 21, 04103 Leipzig, Tel.: 0341-9715898, sven_r@yahoo.com

Comment 1 ET16_purified
Comment 2 MW: av 3155,8 (M+H)+

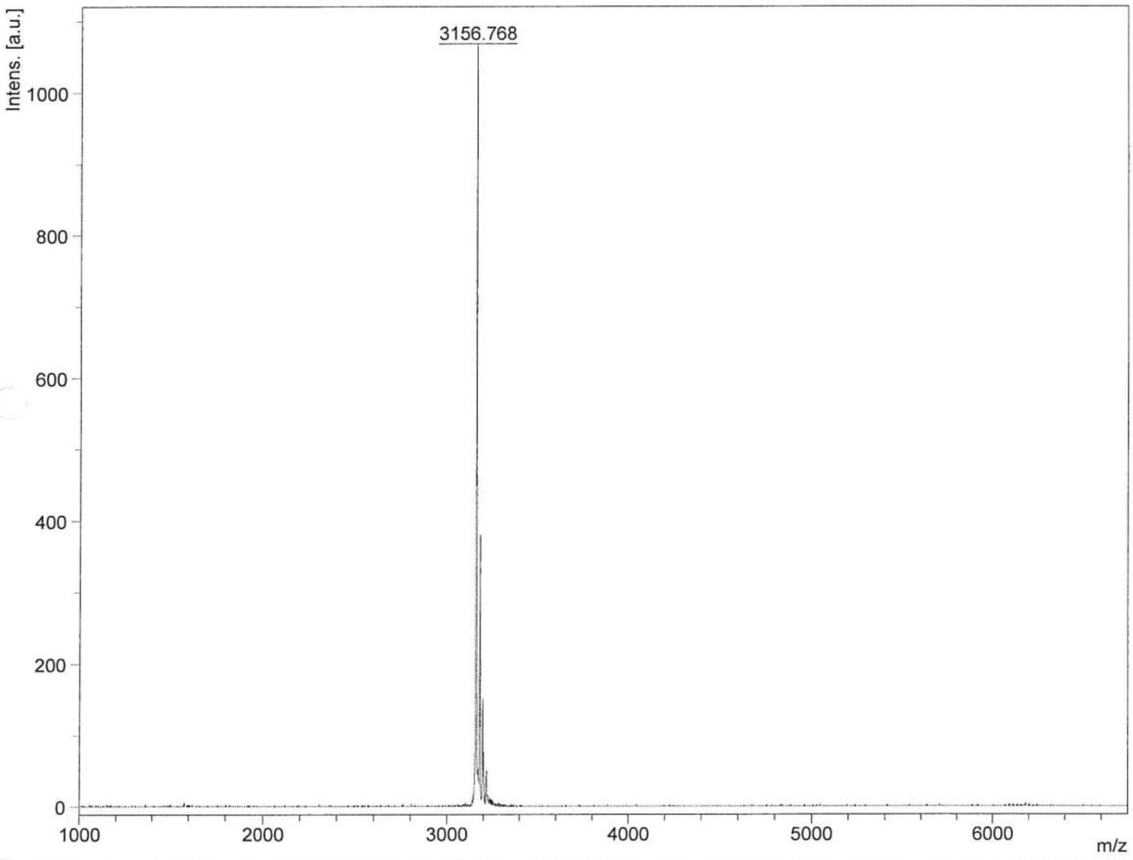

Acquisition Parameter

Date of acquisition	2017-01-04T11:31:48.981+01:00
Acquisition method name	D:\Methods\flexControlMethods\LP_PepMix.par
Aquisition operation mode	Linear
Voltage polarity	POS
Number of shots	100
Name of spectrum used for calibration	
Calibration reference list used	PeptideCalibStandardInsulin2 monoAv

Instrument Info

User	IZKF
Instrument	FLEX-PC
Instrument type	microflex

D:\Data\IZKF2016\ET16_purified\0_E12\1 printed: 1/4/2017 11:33:46 AM

119 **IZKF Leipzig, Core Unit Peptid-Technologien**

Liebigstraße 21, 04103 Leipzig, e-mail: sven_r@yahoo.com, Tel.: 0341 - 9715898 / 897

Comment:

Sample Name:	**AD23 purified**	*Injection Volume:*	**20.0**
Vial Number:	**GE1**	*Channel:*	**UV_VIS_1**
Sample Type:	**unknown**	*Wavelength:*	**220.0**
Control Program:	**Peptide_3D_basic_short**	*Bandwidth:*	**4**
Quantif. Method:	**peptide_izkf**	*Dilution Factor:*	**1.0000**
Recording Time:	**11/4/2023 12:54**	*Operator:*	**KEYUSER**
Sample ID:		*Sample Amount:*	**1.0000**

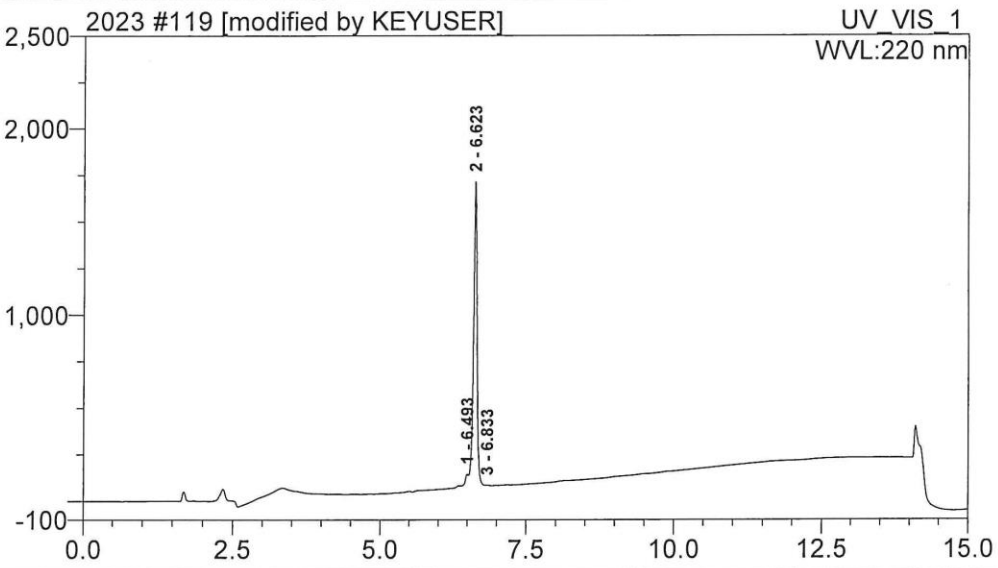

No.	Ret.Time min	Peak Name	Height mAU	Rel.Area %	Area mAU*min	Amount	Type
1	6.49	n.a.	65.0	2.99	3.55	n.a.	BM *
2	6.62	n.a.	1633.2	96.85	114.82	n.a.	M *
3	6.83	n.a.	3.1	0.16	0.19	n.a.	MB*
**Total:**			1701.326	100.000	118.55	0.000	

Chromeleon (c) Dionex 1996-2006
Version 6.80 SR15 Build 4656 (243203)

Dr. Sven Rothemund, IZKF Leipzig, Liebigstrasse 21, 04103 Leipzig, Tel.: 0341-9715898, sven_r@yahoo.com

Comment 1 AD23 purified
Comment 2 MW:4321,6(M+H)+

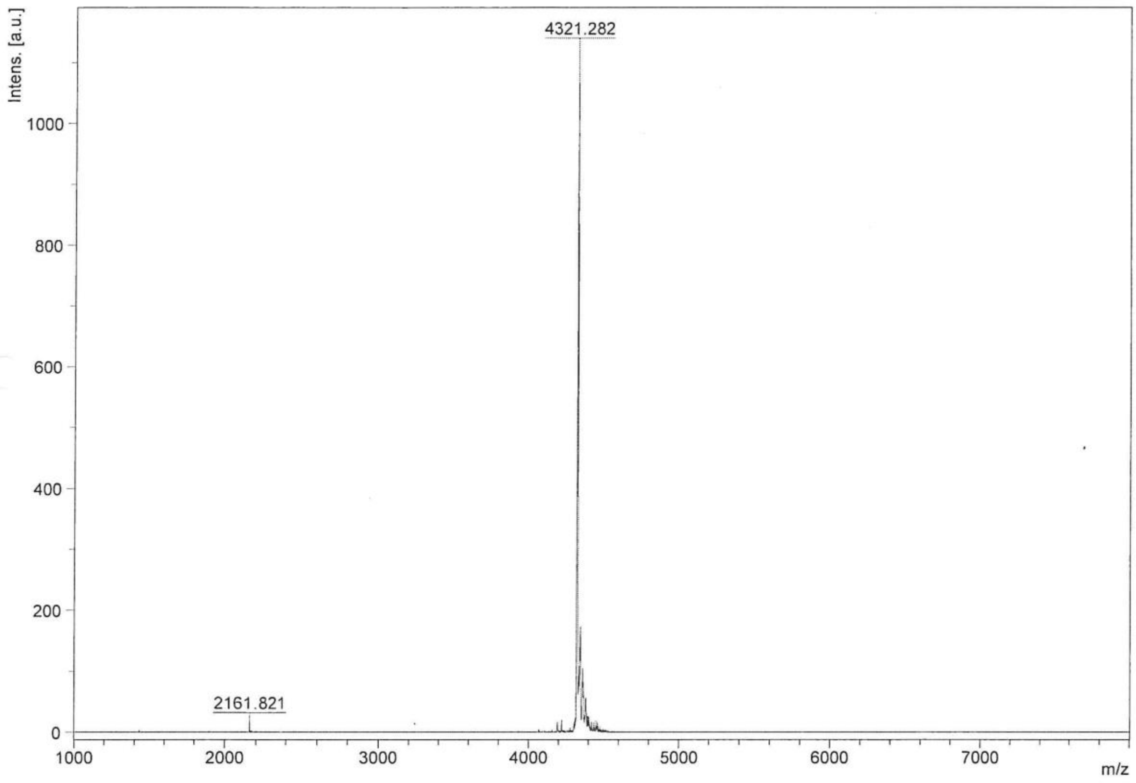

quisition Parameter

Date of acquisition	2023-04-11T12:17:59.143+02:00
Acquisition method name	D:\Methods\flexControlMethods\LP_PepMix.par
Aquisition operation mode	Linear
Voltage polarity	POS
Number of shots	75
Name of spectrum used for calibration	
Calibration reference list used	PeptideCalibStandardInsulin2 monoAv

Instrument Info

User	IZKF
Instrument	FLEX-PC

D:\Data\IZKF2023\AD23 purified\0_B9\1 printed: 11.04.2023 13:22:06

127 IZKF Leipzig, Core Unit Peptid-Technologien

Liebigstraße 21, 04103 Leipzig, e-mail: sven_r@yahoo.com, Tel.: 0341 - 9715898 / 897

Comment:

Sample Name:	**AE23 purif**	*Injection Volume:*	**30.0**
Vial Number:	**BE1**	*Channel:*	**UV_VIS_1**
Sample Type:	**unknown**	*Wavelength:*	**220.0**
Control Program:	**Peptide_3D_basic_short**	*Bandwidth:*	**4**
Quantif. Method:	**Peptide_3D_basic**	*Dilution Factor:*	**1.0000**
Recording Time:	**14/4/2023 14:33**	*Operator:*	**KEYUSER**
Sample ID:		*Sample Amount:*	**1.0000**

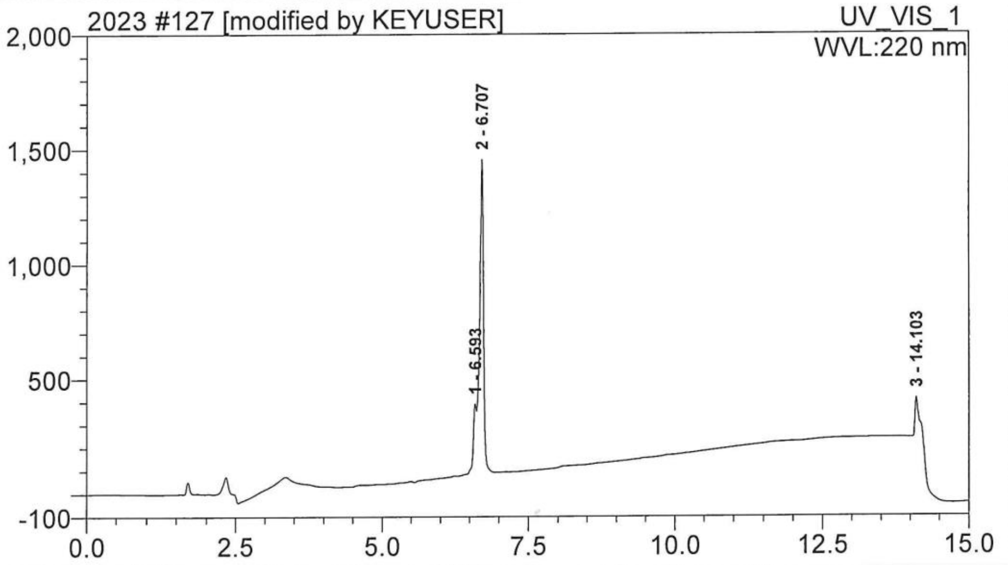

No.	Ret.Time min	Peak Name	Height mAU	Rel.Area %	Area mAU*min	Amount	Type
1	6.59	n.a.	292.2	1.15	14.29	n.a.	BM *
2	6.71	n.a.	1356.3	8.59	106.61	n.a.	MB*
3	14.10	n.a.	447.2	90.26	1120.31	n.a.	BMB
**Total:**			2095.738	100.000	1241.21	0.000	

Chromeleon (c) Dionex 1996-2006
Version 6.80 SR15 Build 4656 (243203)

Dr. Sven Rothemund, IZKF Leipzig, Liebigstrasse 21, 04103 Leipzig, Tel.: 0341-9715898, sven_r@yahoo.com

Comment 1 AE23 purified
Comment 2 MW: 4118

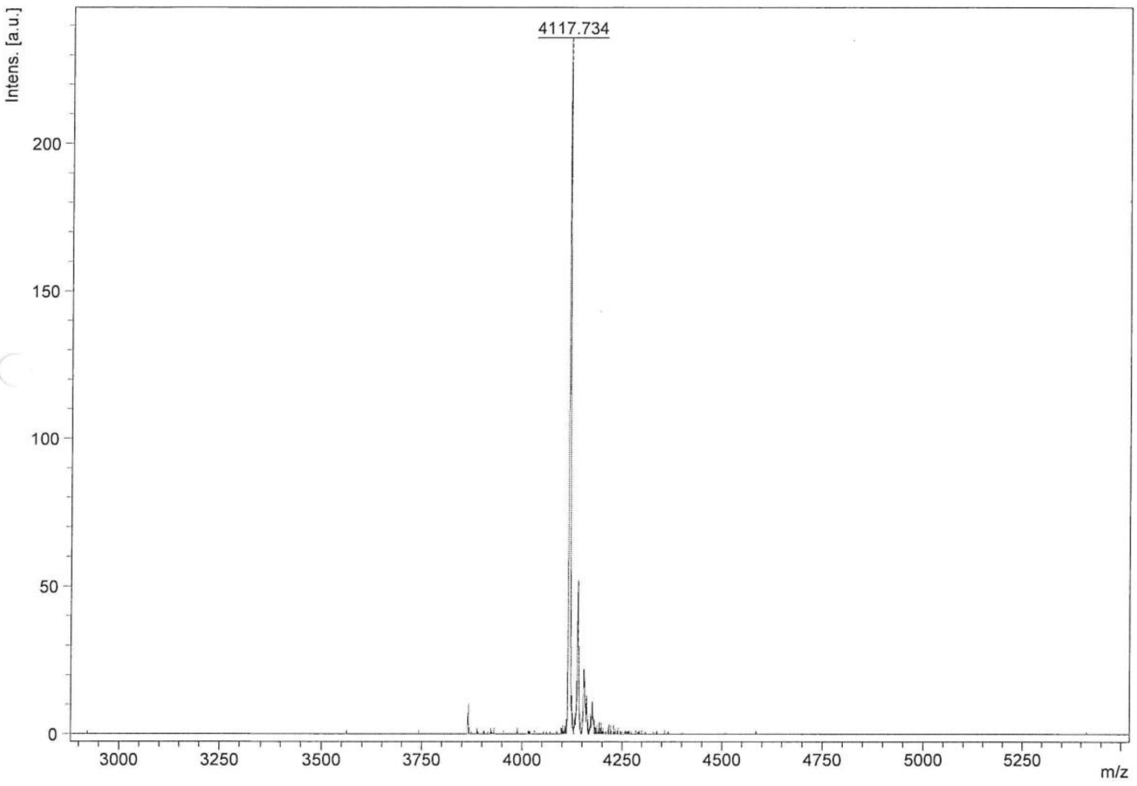

uisition Parameter

Date of acquisition	2023-04-14T10:51:24.740+02:00
Acquisition method name	D:\Methods\flexControlMethods\LP_PepMix.par
Aquisition operation mode	Linear
Voltage polarity	POS
Number of shots	61
Name of spectrum used for calibration	
Calibration reference list used	PeptideCalibStandardInsulin2 monoAv

Instrument Info

User	IZKF
Instrument	FLEX-PC

D:\Data\IZKF2023\AE23 purif\0_C12\1 printed: 14.04.2023 10:52:15

133 IZKF Leipzig, Core Unit Peptid-Technologien
Liebigstraße 21, 04103 Leipzig, e-mail: sven_r@yahoo.com, Tel.: 0341 - 9715898 / 897

Comment:

Sample Name:	**AF23 purified**	*Injection Volume:*	**20.0**
Vial Number:	**RA1**	*Channel:*	**UV_VIS_1**
Sample Type:	**unknown**	*Wavelength:*	**220.0**
Control Program:	**Peptide_3D_basic_short**	*Bandwidth:*	**4**
Quantif. Method:	**peptide_izkf**	*Dilution Factor:*	**1.0000**
Recording Time:	**18/4/2023 11:34**	*Operator:*	**KEYUSER**
Sample ID:		*Sample Amount:*	**1.0000**

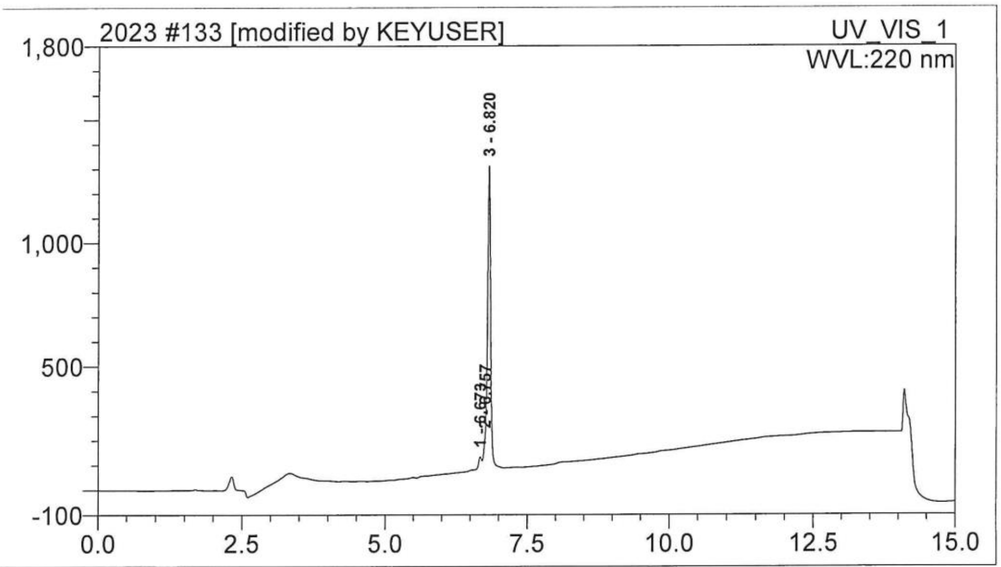

No.	Ret.Time min	Peak Name	Height mAU	Rel.Area %	Area mAU*min	Amount	Type
1	6.67	n.a.	41.8	2.63	2.18	n.a.	BM *
2	6.76	n.a.	114.5	3.66	3.03	n.a.	M *
3	6.82	n.a.	1213.6	93.71	77.61	n.a.	MB*
**Total:**			1369.855	100.000	82.82	0.000	

Chromeleon (c) Dionex 1996-2006
Version 6.80 SR15 Build 4656 (243203)

Dr. Sven Rothemund, IZKF Leipzig, Liebigstrasse 21, 04103 Leipzig, Tel.: 0341-9715898, sven_r@yahoo.com

Comment 1	AF23 purified
Comment 2	MW:3125,3(M+H)+

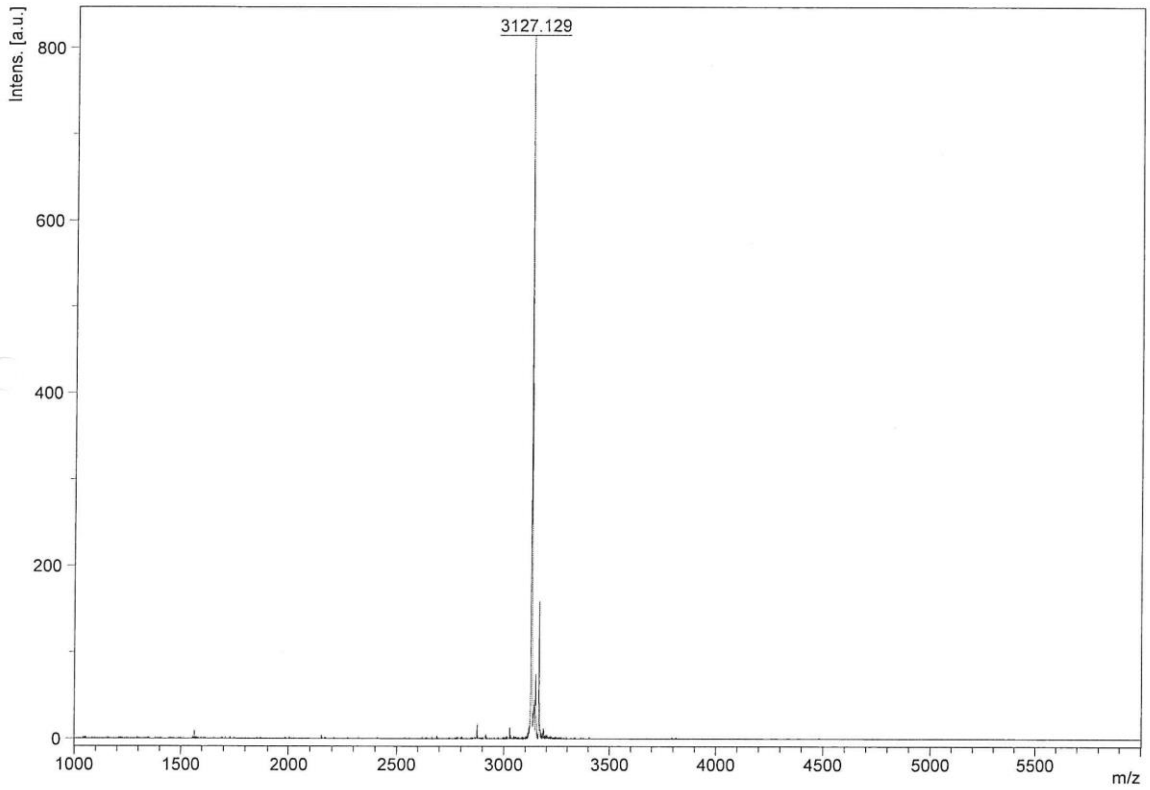

quisition Parameter

Date of acquisition	2023-04-18T12:03:01.569+02:00
Acquisition method name	D:\Methods\flexControlMethods\LP_PepMix.par
Aquisition operation mode	Linear
Voltage polarity	POS
Number of shots	100
Name of spectrum used for calibration	
Calibration reference list used	PeptideCalibStandardInsulin2 monoAv

Instrument Info

User	IZKF
Instrument	FLEX-PC

D:\Data\IZKF2023\AF23 purified\0_H10\2 printed: 18.04.2023 14:22:12
